# Adaptive developmental plasticity: Compartmentalized responses to environmental cues and to corresponding internal signals provide phenotypic flexibility

**DOI:** 10.1186/s12915-014-0097-x

**Published:** 2014-11-21

**Authors:** Ana Rita A Mateus, Manuel Marques-Pita, Vicencio Oostra, Elvira Lafuente, Paul M Brakefield, Bas J Zwaan, Patrícia Beldade

**Affiliations:** Instituto Gulbenkian de Ciência, Rua da Quinta Grande 6, P-2780 Oeiras, Portugal; Institute of Biology, Leiden University, Sylviusweg 72, 2333 BE Leiden, The Netherlands; School of Informatics and Computing, Indiana University, 919 East Tenth Street, Bloomington, IN 47408 USA; Department of Zoology, Cambridge University, Downing Street, Cambridge, CB2 3EJ UK; Laboratory of Genetics, Wageningen University, Droevendaalsesteeg 1, 6708 PB Wageningen, The Netherlands

**Keywords:** *Bicyclus anynana*, Developmental recombination, Ecdysone, Environmental input, Modularity, Phenotypic flexibility, Physiology, Seasonal polyphenism, Thermal plasticity, Trait-specific sensitivities

## Abstract

**Background:**

The environmental regulation of development can result in the production of distinct phenotypes from the same genotype and provide the means for organisms to cope with environmental heterogeneity. The effect of the environment on developmental outcomes is typically mediated by hormonal signals which convey information about external cues to the developing tissues. While such plasticity is a wide-spread property of development, not all developing tissues are equally plastic. To understand how organisms integrate environmental input into coherent adult phenotypes, we must know how different body parts respond, independently or in concert, to external cues and to the corresponding internal signals.

**Results:**

We quantified the effect of temperature and ecdysone hormone manipulations on post-growth tissue patterning in an experimental model of adaptive developmental plasticity, the butterfly *Bicyclus anynana*. Following a suite of traits evolving by natural or sexual selection, we found that different groups of cells within the same tissue have sensitivities and patterns of response that are surprisingly distinct for the external environmental cue and for the internal hormonal signal. All but those wing traits presumably involved in mate choice responded to developmental temperature and, of those, all but the wing traits not exposed to predators responded to hormone manipulations. On the other hand, while patterns of significant response to temperature contrasted traits on autonomously-developing wings, significant response to hormone manipulations contrasted neighboring groups of cells with distinct color fates. We also showed that the spatial compartmentalization of these responses cannot be explained by the spatial or temporal compartmentalization of the hormone receptor protein.

**Conclusions:**

Our results unravel the integration of different aspects of the adult phenotype into developmental and functional units which both reflect and impact evolutionary change. Importantly, our findings underscore the complexity of the interactions between environment and physiology in shaping the development of different body parts.

**Electronic supplementary material:**

The online version of this article (doi:10.1186/s12915-014-0097-x) contains supplementary material, which is available to authorized users.

## Background

In numerous species, the external environment can affect development and lead to the production of distinct phenotypes from the same genotype [1]. This phenomenon is called developmental plasticity. The resulting alternative phenotypes can be as dramatically different as the nutrition-induced differences between workers and queens in social insects (for example, [2-4]) and the seasonal forms of many insects (for example, [5-7]). All organisms have traits that are plastic. However, not all body parts of plastic organisms are equally flexible (for example, [8-10]). The ability of tissue development to both resist and integrate environmental input is crucial for organismal fitness in heterogeneous environments. An important step towards understanding how organisms can adaptively respond to the environment by expressing alternative phenotypes, and organize this response across body parts and traits, is to determine to which degree and by what mechanism body parts are integrated into coordinated modules that correspond to functional, evolutionary and/or developmental units [11,12]. This will include understanding how different body parts respond to external environmental cues, as well as to the internal signals that convey information about those cues to the developing tissues.

In insects, ecdysteroid hormones work as internal signals that mediate key developmental transitions, such as molting and metamorphosis, and can also mediate developmental plasticity [7]. The external environment typically affects systemic hormone titers which, in turn, affect developing tissues. So that different traits which respond to the same hormone signal can develop and evolve independently, hormone effects need to be compartmentalized in time and space [7,13]. This type of compartmentalization has been characterized in relation to the environmental regulation, mostly by nutrition, of the growth of different organs during insect larval development [10,14,15]. Much less is known about the compartmentalization of hormone effects for different groups of cells within the same tissue, and during post-growth tissue patterning. We investigate this process here for an evolutionary ecology model of developmental plasticity.

**Figure 1:**
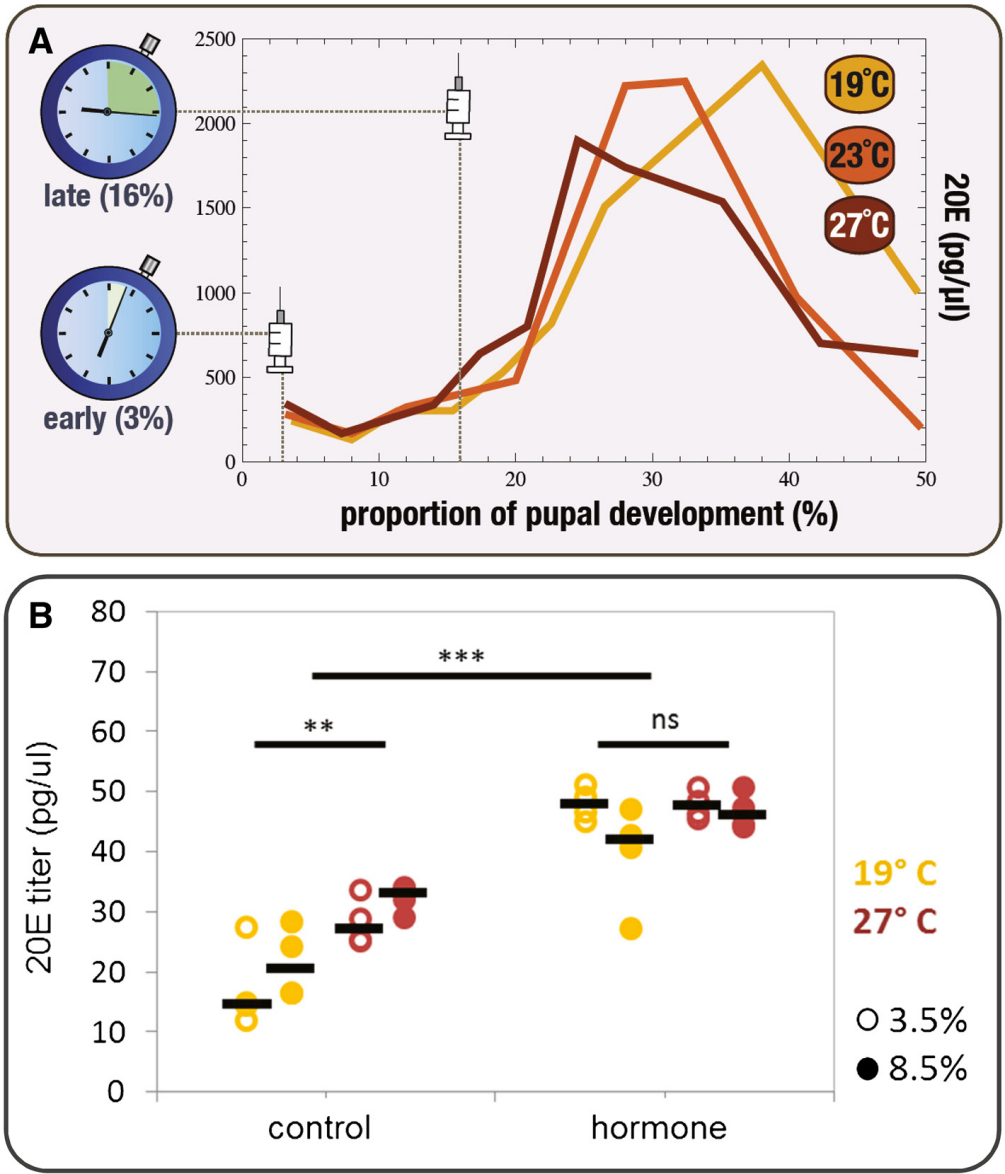
**Dynamics and manipulation of internal levels of ecdysone. (A)** Experimental design for hormone manipulations. Hydroxyecdysone (20E) injections were done on female pupae reared at 19°C, 23°C or 27°C at two developmental stages corresponding to different phases of the natural 20E dynamics (compare with [21]): ‘early’, before ecdysone concentration starts to increase (at 3% of the total time it takes to complete pupal development at each of the temperatures), and ‘late’, corresponding to the ascending phase of the ecdysone level (at 16% of the total pupal development time). **(B)** Effect of early hormone injections on hormone titers. Internal levels of 20E at 3.5% and 8.5% of total pupal development time after early injection of hormone and control solutions at 19°C and 27°C. The bar represents the median value of four individuals per treatment, temperature, and time point (see Material and methods). We tested for the effect of temperature and injection treatment on the levels of 20E at two time points using the model 20E ~ time point + temperature * injection, for which the residuals showed no significant departure from normality (Shapiro-Wilk W test: *W* = 0.950, *P* = 0.146) or from homogeneity of variances (Fligner-Killeen test: *Median Chi Square* = 1.176, *df* = 1, *P* = 0.185). The analysis of variance revealed a statistically significant effect of *temperature* (*F*_*(1,32)*_ = 13.848, *P* = 0.0009) and *injection* (*F*_*(1,32)*_ = 114.501, *P* = 3.25e-11), but not of *time point* (*F*_*(1,32)*_ = 0.026, *P* = 0.874) or temperature*injection (*F*_*(1,32)*_ = 3.670, *P* = 0.066). Results of the *post-hoc* pairwise comparisons using the Tukey honest significance test are indicated in the figure: *ns* for *P* >0.01, ** for *P* <0.01, *** for *P* <0.001 [see Additional file 4 for more on this analysis].

The butterfly *Bicyclus anynana* has become a textbook example of adaptive developmental plasticity [1,16-19]. Its study combines knowledge about the ecological and evolutionary significance of plasticity with the analysis of its genetic and physiological underpinnings [1,20]. In natural populations, butterflies developing in the dry versus the wet season have cryptic versus conspicuous ventral wing patterns, each associated with different seasonal strategies to avoid predation [1]. The wing phenotypes encompass a whole suite of pattern elements which differ between the seasons. In the laboratory, the development of wet- versus dry-like phenotypes can be induced by the temperature experienced during pre-adult stages [1]: warmer temperatures induce wet-like wing patterns, while cooler temperatures induce dry-like phenotypes. Previous studies showed differences between warm- versus cool-reared pupae in the dynamics of ecdysone levels [21] (Figure 1A) and established these as a cause for changes in wing pattern [21]. Various studies of *B. anynana* wing pattern plasticity characterized the effects of the temperature and/or ecdysteroid levels on a few indicative pattern traits [22-25]. Limiting these analyses to only a few traits has precluded an assessment of how the effects of external and internal signals are compartmentalized in the developing wings. A systematic analysis of both types of cues on multiple aspects of wing patterns is lacking.

**Figure 2:**
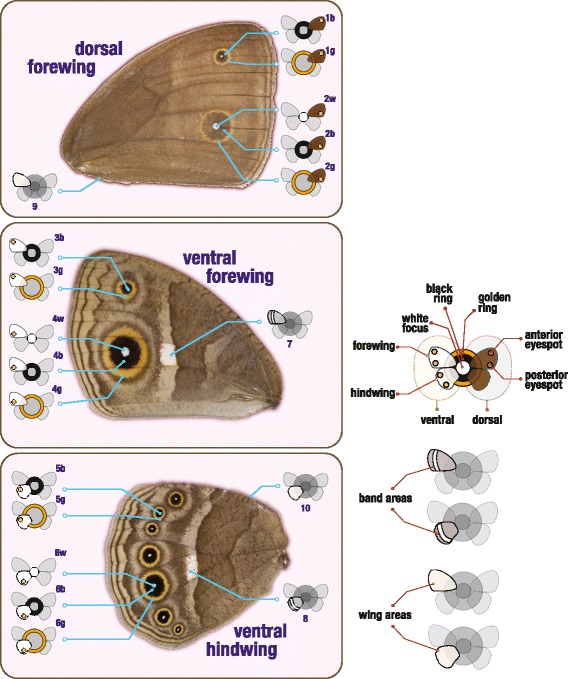
**Wing traits measured in adult females.** The photos represent the typical phenotype of female *Bicyclus anynana* reared at 27°C. Note that the dorsal surface of the hindwing does not always have color patterns beyond occasional extra eyespots or just their white pupils which are generally too small for accurate size measurements. For each individual, we obtained 19 wing measurements corresponding to four categories of traits: dorsal eyespots, ventral eyespots, ventral band and wing areas. Note that each eyespot corresponds to a different trait number and we use different letter codes to refer to the corresponding white centers (w), black discs (b) and golden rings (g). The diagram on the right panel displays the symbols used to refer to each of the traits in the other figures. On each wing surface (ventral represented in white and dorsal in brown), we measured two eyespots (one more anterior represented by a circle on the top and one more posterior by a circle on the bottom). The color of the circles at the center of the image corresponds to each of the three color rings that make up each eyespot.

To characterize the effects of external cues and internal signals on tissue patterning, we manipulated temperature during pre-adult development and manipulated the levels of active ecdysone in the pupal hemolymph (Figure 1). We then compared the suite of adult wing traits that constitute the seasonal wing phenotype. The traits we chose (Figure 2) reflect increasing levels of spatial resolution in the analysis of the compartmentalization of plasticity. They allow comparisons between: 1) different wings derived from autonomously-developing imaginal discs (fore- and hindwing); 2) different surfaces of the same wing that correspond to distinct cell sheets (dorsal and ventral surfaces) and evolve under different selection regimes [26]; 3) different types of pattern elements (eyespots and band) displaying weak genetic correlations between them; 4) different repeats of the same type of pattern element (anterior and posterior eyespots on the same wing surface) with stronger correlations between them [19,27]; and 5) different rings of the same eyespot (central white focus, middle black disc, and external golden ring) that correspond to groups of neighboring cells responding to a morphogen signal originated at each presumptive eyespot center [19,28-31]. Our data on this extensive set of traits allow us to investigate the coordination of responses to external cues and internal signals across groups of wing epidermal cells and the mechanism for the spatial compartmentalization of the sensitivities to those signals. We discuss our results in terms of whether tighter or looser integration between traits might be adaptive and/or might represent (constrained) properties of the development in response to environmental variation.

## Results and discussion

**Figure 3:**
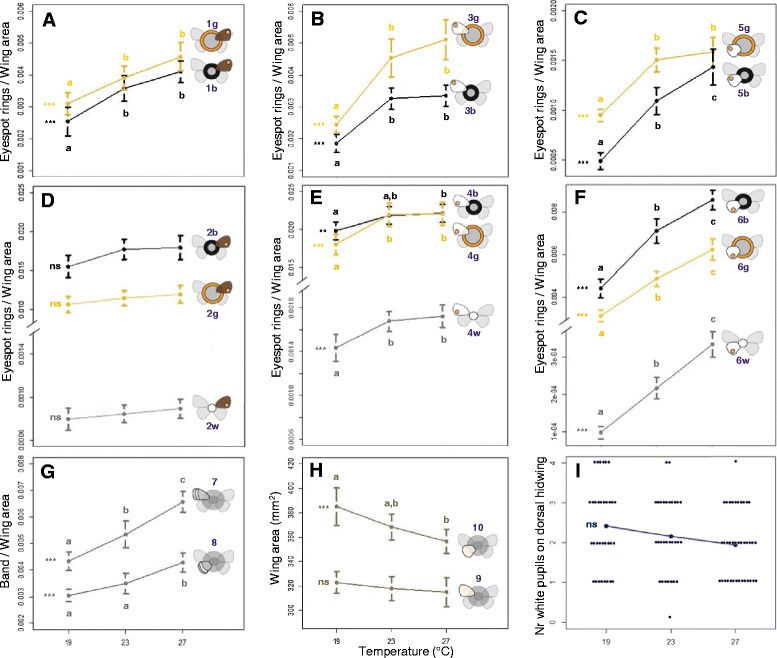
**Effect of temperature experienced during development on wing traits.** For each trait, we plot the mean value as a function of temperature and use bars to represent the standard deviation for 24 to 38 measurements per temperature. These representations, called reaction norms, are the standard way of displaying plasticity. Trait icons, compare with Figure 2, are given on the right of the respective reaction norm line: **(A-B)** dorsal eyespots, **(C-F)** ventral eyespots on forewing and hindwing, **(G)** ventral bands and **(H)** wing areas. We tested for the effect of temperature on wing pattern trait using the model *trait ~ temperature + wing* (where the area of the corresponding *wing* is a covariate) and on wing area using *wing ~ temperature* (see Material and methods). Trait values were used untransformed or log10 transformed to meet the Shapiro-Wilk normality test (alpha = 0.05). Statistical significance for effects of temperature on wing traits (see Material and methods) is indicated to the left of each reaction norm: ns (non-significant), *P >0.01*, **P <0.01, ***P <0.001. When ANCOVA/ANOVA showed significant effects of temperature on trait value, we compared across temperatures. For each reaction norm, different letters indicate pairwise comparisons that revealed statistically significant differences (lsmeans *P <0.01*) (see Additional file 1 for more details on these statistical analyses). For the number of white pupils (n = 30 to 38 individuals, Additional file 1) on the dorsal surface of the hindwing in panel **(I)**, we found no significant effect of temperature using the model *pupil nr ~ temperature* with a quasi-Poisson distribution (Deviance = 1.894, *df* = 2, *P* = 0.1172). ANCOVA, analysis of covariance; ANOVA, analysis of variance.

**Figure 4:**
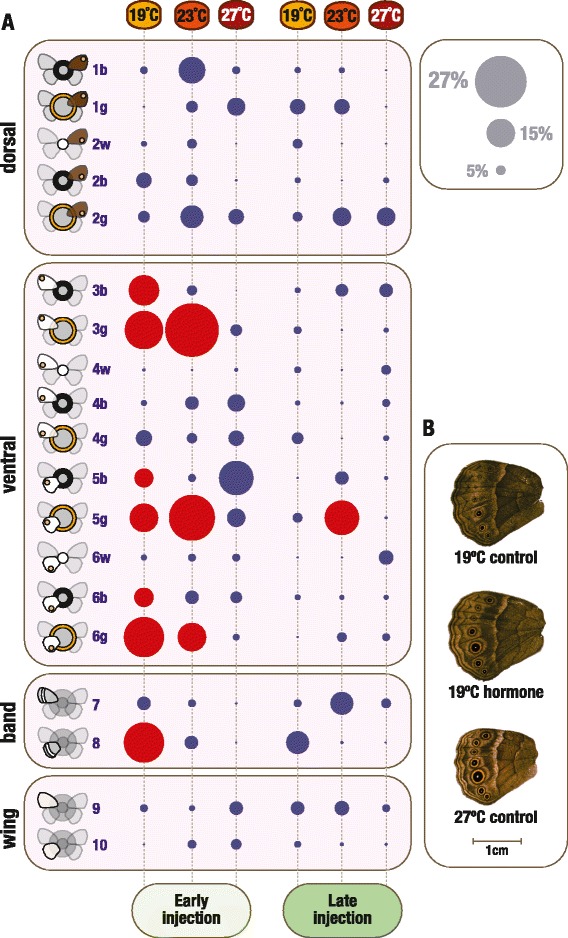
**Effect of pupal hormone manipulations on different wing traits. (A)** For each trait, temperature and time point combination, the circles represent the magnitude (circle size; scale on top right corner) and statistical significance (circle color, with red for significant differences; compare with permutation test explained in the Material and methods) of the difference between hormone- versus control-injected individuals (details in Additional file 2). As for Figures 2 and 3, the traits are organized per type: dorsal eyespot traits, ventral eyespot traits, ventral bands and wing areas. The final number of measurements for each trait in each experimental group can be found in Additional file 2. The difference between control and hormone treatments was tested using a series of core and confirmatory statistical tests, all giving largely the same results (details in Materials and methods and Additional file 2). **(B)** Photos of the ventral surface of adult hindwings representing the phenotypes of different temperature and injection treatments: control-injected individual at 27°C, hormone-injected individual at 19°C and control-injected individual at 19°C. Scale bar corresponds to 1 cm. All images are from butterflies injected as pupae at 3% of their development time. These wings illustrate how early hormone manipulations at lower temperature increase the area of different color pattern components, bringing the phenotypes closer to those of individuals reared at higher temperature.

**Figure 5:**
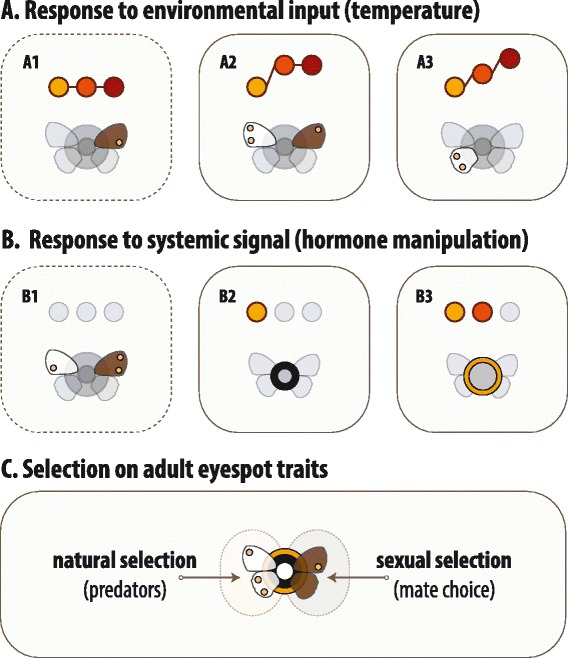
**Patterns of coordinated response to external and internal signals.** Each box includes eyespot traits that responded in a similar manner to differences in developmental temperature **(A)** and to hormone injections **(B)**. Boxes in dashed lines represent traits (symbols, compare with Figure 2) that do not respond to temperature (A1) or to hormone injections (B1). The other boxes represent distinct patterns of response to temperature (A2-A3) or to ecdysone (B2-B3) [see details in Additional file 5]. The three circles at the top of each box represent each of the three experimental temperatures: from right to left, 19°C, 23°C and 27°C. In panel **(A)**, lines between those circles illustrate the shapes of the corresponding thermal reaction norms (compare with Figure 3): flat for A1, 19°C <23°C approximately 27°C for A2, and 19°C <23°C <27°C for A3. In panel **(B)**, the circles not in gray represent temperatures for which phenotypes were significantly different between control- and hormone-injected individuals (compare with Figure 4): no effect of hormone manipulations for whichever temperature in B1, effect only for 19°C in B2 and effect both at 19°C and 23°C in B3. The only traits that do not respond to temperature (A1) correspond to the eyespot shown to be under sexual selection, while those that do not respond to hormone manipulations (B1) are those not exposed to predators in resting butterflies **(C)**. The patterns of response to temperature contrast fore- and hindwing while those for hormone manipulations contrast black and golden color rings. A detailed scheme of the patterns of response showing all traits can be found in Additional file 5.

**Figure 6:**
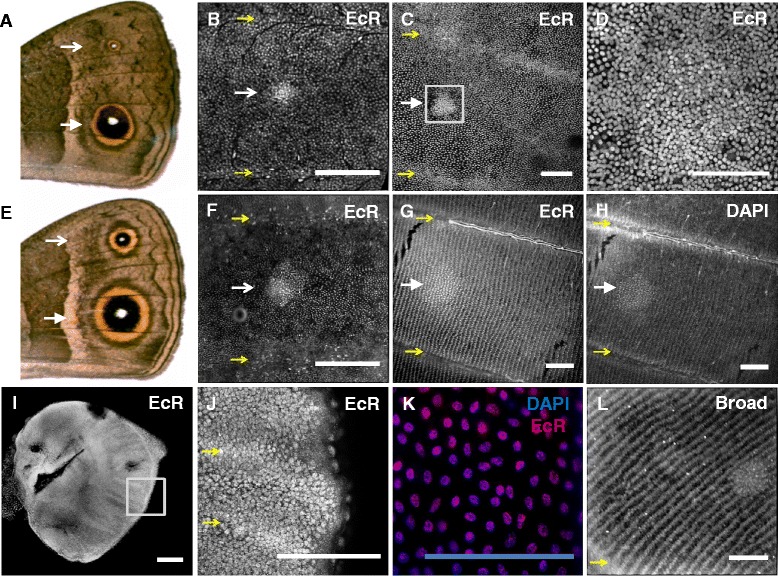
**Localization of ecdysone receptor (EcR) protein in larval wings. (A)** Ventral surface of forewing (distal section shown) of non-injected individual reared at 19°C with different arrow heads pointing at anterior (trait 3) versus posterior (trait 4) eyespots. Corresponding region of anterior **(B)** and posterior **(C)** eyespot fields of developing pupal forewing at 19°C around 6% and 23% of pupal time, respectively. Panel **(D)** is a detail of the presumptive eyespot center in panel **(C)**. **(E)** Ventral surface of forewing (distal section) of non-injected individual reared at 27°C with arrow heads pointing at anterior and posterior eyespots. Corresponding region of anterior **(F)** and posterior **(G)** eyespot fields of developing pupal forewing at 27°C around 6% and 23% of pupal time, respectively. Panel **(H)** corresponds to the DAPI (nuclear) stain in panel **(G)** showing higher density and lack of row-like organization of the cells at the center of the presumptive eyespot. Panel **(I)** corresponds to EcR expression in larval hindwing and **(J)** is a detail of (I). **(K)** Detail of overlap in EcR protein and DAPI from developing forewing at 27°C (around 6% of pupal duration), showing nuclear localization of EcR. **(L)** Presumptive eyespot center (around 23% of pupal duration at 27°C) expressing EcR’s target gene Broad (core isoform) shows that EcR is active. Yellow arrows indicate veins for reference. All in all, we see EcR-positive cells over the entire wing from larval to late pupal stages, and in higher cell density in the presumptive eyespot centers. These centers are larger for larger eyespots. Scale bar = 100 μm. DAPI, 4′,6-diamidino-2-phenylindole.

Our results show that different groups of cells on the developing wing epidermis, which correspond to different aspects of the color pattern on adult female wings, have characteristic sensitivities to changes in temperature during pre-adult development (Figure 3), as well as to changes in ecdysone levels during the pupal stage (Figure 4). We could identify not only which traits are, and are not, responsive to manipulations of the external cue and internal signal, but also identify groups of sensitive traits that display distinct patterns of coordinated responses (Figure 5). Finally, we show that the spatial compartmentalization of hormone sensitivities is not due to the spatial or temporal compartmentalization of the hormone receptor protein (Figure 6).

### Response of wing traits to developmental temperature

To assess how different groups of cells on the developing wings respond to external environmental cues, we measured wing patterns of butterflies reared at three temperatures, representing typical wet- and dry-inducing extremes (27°C and 19°C, respectively) and an intermediate temperature (23°C). We then compared phenotypes between temperatures. Figure 3 shows the thermal reaction norms for the 19 target traits in adult females. For the first time, this involved considering separately and simultaneously the distinct color rings (white, black, and gold) of multiple eyespots on different parts (anterior and posterior) of the same wing surface and on different wing surfaces (ventral and dorsal) (Figure 2).

This extensive analysis of wing pattern traits revealed that, in contrast to what had been described, some aspects of the dorsal wing pattern are plastic in relation to developmental temperature (Figure 3A). Previous studies of plasticity on dorsal forewing color pattern had investigated the most posterior eyespot (our trait 2) and found it to be largely non-plastic across seasonal environments [22,25]. Our results confirm this but, by also analyzing other pattern elements on the same wing surface, show that the lack of temperature-sensitivity is not a property of the whole dorsal wing surface. The more anterior eyespot on the dorsal forewing (trait 1) did increase significantly with temperature (Figure 3A). As expected from previous studies, wing pattern components on the ventral surface of the wings showed clear thermal plasticity (Figure 3B, C, E, F, G; see Additional file 1).

### Only the wing pattern element implicated in mate choice does not respond to temperature

Previous work largely focused on ventral wing patterns because this is the surface exposed to predators in butterflies at rest and, thus, the surface under predator-driven natural selection for plasticity [20]. Seasonal variation in ventral wing patterns is associated with seasonal variation in the resting background and to alternative strategies for butterflies to avoid predation. In the cooler dry season, duller brown wing patterns with no striking color elements are cryptic in relation to the resting background of dry brown leaves. In the warmer wet season, more conspicuous color elements along wing margins can function as targets for predator attacks away from the more fragile body [1,32].

The dorsal patterns, on the other hand, are typically not exposed in the butterfly at rest and presumably not under selection by predators. Instead, those patterns are exposed during courtship and thought to evolve under sexual selection [25,26,33]. In particular, some of the UV-reflecting white pupils of dorsal eyespots have been shown to influence mate choice [25,34]. In our study of female butterflies, the only eyespot that showed no significant response to temperature (Figure 3D; trait 2) was the one that is sexually selected in males [25]. The white center of this eyespot had been found to be plastic in males; being larger and more UV-reflecting in wet season courting individuals [25]. Even though it has been proposed that dry season females do courtship [25], in a case of seasonally-plastic sexual selection, we found that the corresponding trait is not plastic in females (Figure 3D; trait 2w). Instead, a recent study proposed that male choice among potential dry-season mating partners depends on the number of white pupils found on the dorsal surface of the female hindwing [34]. The number of such pupils was shown to vary between females reared at 17°C versus 27°C [34]. In our study, we found that the mean (but not the median) number of white pupils on the ventral surface of the hindwing of non-injected females decreases with increasing temperature, but not significantly so (Figure 3I).

### Response of wing traits to hormone manipulations

To examine how different groups of cells on the wings respond to changes in hormone levels, we measured the effect of hormone manipulations during the early pupal stage when the signaling from eyespot organizers and the response of the surrounding cells to the ring-determining morphogen are known to take place [19]. We manipulated the levels of active ecdysone in the hemolymph by injecting female pupae with 20-hydroxyecdysone (20E) [22-24] at two developmental time points (Figure 1). For each temperature and injection time point, we then compared adult wings between control-injected and hormone-injected individuals. Figure 4 shows the magnitude and statistical significance of the difference between control and hormone treatments for each of the target traits, injection time points, and rearing temperatures [see also Additional file 2].

Only traits that responded to changes in temperature during development responded to changes in hormone titers during early pupal life. That is, all traits for which differences between control-injected and hormone-injected individuals were significant (that is, any red circles in Figure 4) are traits for which the differences between temperatures for non-injected individuals were also significant (that is, reaction norms marked with stars in Figure 3). However, not all wing pattern traits that responded to temperature were affected by the hormone treatment. We found no significant effect of hormone manipulations for any of the traits in the dorsal wing surface (Figure 4A). In contrast, many traits on the temperature-plastic ventral wing surfaces significantly increased in area in response to hormone injections. In some cases, lack of effect of our hormone injections on temperature-responsive traits can be explained by the fact that trait determination occurred before the hormone treatment. This is the case for the white eyespot centers (traits 4w, 6w in Figure 2) and for hindwing area (trait 10). The establishment of the eyespot organizing centers [35] and most of wing growth [36] are known to take place during larval life, before our hormonal injections were done. However, for other non-responsive traits, notably eyespot color rings, that is not the case (see below).

### Only pattern elements on the wing surface exposed to predators respond to changes in pupal ecdysone levels

For all dorsal (traits 1 and 2) and some ventral thermally-responsive color pattern elements (traits 4 and 7) that did not respond to hormone treatment, it seems unlikely that our treatment missed the relevant windows of trait determination. Certainly for eyespot rings, we know that it is during early pupal development that signaling from eyespot centers establishes concentric rings of cells fated to produce different color pigments [30,31]. The lack of response of those traits to our hormone manipulations could be due to lower sensitivities to hormone titers and due to them requiring hormone concentrations higher than those we produced artificially. This, too, at least alone, seems unlikely because our post-injection hormone levels at 19°C surpassed the control levels at 27°C, a temperature difference for which the traits did change (see below and Figure 1B). The lack of response to hormonal manipulations suggests that thermal plasticity for these traits is not mediated (exclusively) by ecdysone.

It is curious to note that the color traits established in early pupae which we found to be thermally-sensitive but ecdysone-resistant are presumably under no, or weaker, selection by predators. As discussed before, this is the case for color patterns on the dorsal surface of the wing which is not exposed in the butterflies resting against the seasonally color-variable background foliage. Also, unlike other ventral pattern elements, the wing region containing the hormone-unresponsive traits 4 and 7 is typically covered by the hindwing in the resting butterfly. Therefore, these traits too are presumably less exposed to the predators that drive selection for seasonally plastic ventral wing patterns. A weaker selection pressure by natural enemies could explain why these particular traits evolved different levels of plasticity.

### Levels and time windows of sensitivity to hormone manipulations

All traits that responded to hormone injection treatment (Figure 4) were larger in hormone-treated relative to control-treated butterflies. The hormone-induced increase in size is consistent with the temperature plasticity: development at warmer temperatures, associated with an earlier increase in natural 20E titers [21-24] (see Figure 1A), leads to the production of more conspicuous wing patterns with larger areas of non-background color (Figure 3). By artificially increasing hormone levels at the lower temperatures, we induced the production of the same type of phenotypic effect that higher temperatures have on wing patterns (Figure 4B; see also Additional file 3). The fact that the artificial increase in hormone levels phenocopied the temperature effect confirms a role for ecdysteroids at this early-pupal developmental stage in mediating thermal plasticity in wing patterns.

Strikingly, we detected the strongest responses to hormone manipulations for injections done at the early developmental time point, when the natural levels of pupal ecdysone are very low and differences between temperatures were previously undetectable [21], and not for injections at the later time point when hormone titer differences between temperatures are clear (Figure 1). This suggests a window of sensitivity to the hormone between our two injection time points, that is, between 3% and 16% of pupal life. For only one of the target traits (trait 5 g), did we see an effect of later hormone manipulation. This indicates some level of heterochrony in the development of this trait, which appears to have a later window of sensitivity to the hormone. Heterochrony, differences in the developmental times and/or rates, is an important contributor to phenotypic diversification, including for butterfly wing patterns [37,38]. We have shown previously that hormone manipulations at later time points do affect a number of life-history traits [39].

We did not observe significant effects of hormone manipulations at higher temperatures (Figure 4), even if our manipulations did significantly change hormone titers. We measured 20E concentration in the hemolymph of pupae at 3.5% and 8.5% of pupal development time for the two extreme experimental temperatures after early injection of hormone and of control solutions (Figure 1B). Hormone levels are significantly higher for hormone-injected versus control-injected pupae at both rearing temperatures [see Additional file 4]. Control pupae show higher 20E levels when reared at 27°C relative to 19°C, consistent with the relatively faster increase in natural hormone titers that occurs at higher temperatures (Figure 1A). After hormone injection we can no longer detect differences in internal levels between temperatures (Figure 1B).

### Differences in trait associations in response to external and internal cues

Focusing on the eyespot traits that are plastic in relation to temperature and/or to hormone titers, we can identify different categories of response [see Additional file 5 summarized in Figure 5]. The principal component analyses [see Additional file 6], a standard approach for analyses of multidimensional datasets such as ours, identified traits with similar and contrasted responses but not with the same resolution as our analyses of individual traits (compare Figures 3 and 4).

The groups identified based on the response to temperature largely contrast eyespots on the forewing versus hindwing (Figures 3 and 5A). All forewing eyespot traits are significantly smaller at 19°C and do not differ between 23°C and 27°C, while all hindwing eyespot traits significantly increase in size with temperature. In summary, for the effects of temperature on wing patterning, we observed looser integration across autonomously-developing wings and tighter coordination of traits on the same wing. The single hindwing trait (trait 5 g) that responds to temperature in the same manner as all forewing traits (Figure 3 and Additional file 5: Figure S2A) is also the only trait significantly affected by late hormone manipulations (Figure 4). It is unclear what, developmentally or ecologically, might be the uniqueness of this trait.

For the traits that we found to be sensitive to early manipulations of pupal hormone levels, we found a different pattern of coordinated responses. Because 1) color rings of each eyespot are specified by the same morphogen gradient established from each eyespot’s center [19,40], 2) each eyespot center produces morphogen independently of other eyespots [30], and 3) eyespot centers have been shown to have higher levels of ecdysone receptor protein [41], we had hypothesized that all rings of a single eyespot would respond to hormone manipulations in concert and relatively independently from those of other eyespots [19,28]. However, rings of the same color, and not rings of the same eyespot, responded in a similar manner (Figures 4 and 5B). All plastic black rings showed hormone-related changes only at 19°C while all golden rings showed hormone-related changes both at 19°C and 23°C (Figure 4). Among the golden rings, we can further distinguish between those from the anterior versus the posterior-half of the wings. They differ in relation to how much hormone-related change we saw at 19°C versus 23°C (Figure 4, Additional file 5: Figure S2B). This is consistent with studies showing coupling of anterior (and of posterior) portions across wing surfaces [27] and uncoupling of anterior versus posterior eyespots within the same wing surface [28,42].

### Compartmentalization of hormone effects is not explained by hormone receptor localization

As a mechanism for local sensitivities to systemic levels of 20E, we hypothesized that groups of cells that responded differently to 20E manipulations would differ in expression of ecdysone receptor (EcR). To test this hypothesis, we investigated the localization of EcR protein in wings from pupae reared at different temperatures using an antibody against *B. anynana*’s EcR [43]. We found EcR in cells on the entire pupal wing epidermis at all temperatures and throughout the whole early pupal life, extending well after the 16% of developmental pupal time used as our last injection time point (Figure 6). The density of EcR-positive cells was higher in circular regions corresponding to the eyespot organizing centers [41]. These regions were smaller for pupae reared at 19°C relative to 27°C (Figure 6B, C versus 6F, G; [44]), and for smaller versus larger eyespots (Figure 6B, F versus 6C, G).

Surprisingly, however, this pattern of EcR expression was detected both for the highly plastic ventral and the hormone-unresponsive dorsal eyespots. This shows that the non-responsiveness of the dorsal color traits to hormone manipulations cannot be due to the corresponding cells not having the receptor for the systemic signal, as had been previously proposed [22]. Our data also did not reveal visible differences in EcR levels between the regions of the presumptive black versus golden eyespot rings (Figure 6B-D and 6F-G) that showed different sensitivities to the hormone injections (Figure 5). This indicates that differences in the way they respond to hormone manipulations (Figure 5B) must be determined either upstream of the binding of 20E to its receptor in the cell nucleus (for example, cell permeability to hormone) or downstream of that (for example, factors interacting with the activated EcR (compare with [14]).

## Conclusions

Environmental cues can have systemic effects but also localized effects in developing organisms. These are typically mediated by hormone signals in the circulating hemolymph which carry the information about the external environment to the developing tissues. However, not all organs and groups of cells within organs have equal sensitivities to the external cues and internal signals. The compartmentalization of these effects reflects what has been called phenotypic integration to imply tight connections between traits or phenotypic independence to refer to connections that are readily uncoupled (discussed in [13]). The present study identified such differing modes of connections for different aspects of butterfly wing patterns in relation to external temperature and to internal levels of ecdysone. With our systematic analysis of multiple traits in different temperature and hormone contexts (Figures 1 and 2), we have: 1) identified which traits are, and which are not, responsive to temperature during development (Figure 3), and to changes in ecdysone levels in early pupal life (Figure 4); 2) identified which of the sensitive traits respond in concert to each of the cues, and shown that these groupings are not the same for both types of cues (Figure 5); and, finally, 3) revealed that the mechanism for spatial compartmentalization of the responses does not reflect the spatial or temporal compartmentalization of the receptor for the internal signal (Figure 6).

### Overview of the effects of developmental temperature and ecdysone manipulations on plastic wing patterning

We found unexpected differences between sensitivity to temperature and to hormone, both in terms of traits that are responsive versus those that are unresponsive, and also in terms of the traits that respond in a coordinated manner (Figure 5). In relation to the effects of external temperature on wing patterning, we showed that all color traits increase in size with increasing temperature (Figure 3) with the exception of the rings of a single eyespot (Figure 3D; Figure 5A1 and C) previously shown to be under sexual selection in males [25]. Among the temperature-sensitive eyespot traits, we found that all color elements on the forewing respond in the same fashion and differently from all but one color element on the hindwing [see Additional file 5: Figure S2A, summarized in Figure 5A]. The contrast between fore- and hindwing is consistent with the hypothesis that traits on autonomously-developing organs are more loosely integrated than traits on the same organ.

In relation to the effect of increasing hormone levels in early pupal life, we showed that only ventral color patterns, known to be associated with seasonally-plastic strategies for avoiding predators, responded (Figure 4; Figure 5B and C). Among the hormone-responsive eyespot traits, we found that rings of the same color respond in concert and in a pattern distinct from rings of another color [see Additional file 5: Figure S2B, summarized in Figure 5B]. This contrast is not consistent with the hypothesis that all rings of the same eyespot show similar sensitivity to hormone levels because they are all specified by a morphogen gradient originating from the eyespot center expressing hormone receptor [41]. We further show that the spatial compartmentalization of hormone effects is not due to the spatial compartmentalization of the levels of hormone receptor protein (Figure 6), as had been suggested [22]. Overall, our results point to complex interactions between the environmental cues that induce developmental plasticity and the internal signals that carry information about those cues to the developing tissues.

### Sensitivities to external cues and internal signals are shaped by and impact phenotypic evolution

The coordinated trait sensitivities are properties of development that may have been favored by selection; for example, because it is important for fitness that traits change in concert. However, they may also be properties of development that are selectively neutral (that is, it is irrelevant whether or not traits develop in concerted fashion) or even evolutionarily constrained (that is, it could be advantageous for traits to change independently but the way they develop makes that difficult [45]). The integration between traits can be a factor constraining future responses to selection if integrated traits are selected to change in opposite ways (evolutionary constraint hypothesis [13,46]). On the other hand, having traits responding independently to systemic hormone or external input can allow more rapid evolution of new arrangements of traits (evolutionary potential hypothesis [46]). It has been proposed that trait ‘reorganization’ produced by exposure to novel environmental conditions can lead to the production of new phenotypic variants and differences between species, through a process that has been called developmental recombination [47].

To understand fully this type of phenomenon it will be necessary to expand on studies such as ours. It is fundamental to combine the analysis of how different traits are integrated in their response to internal and external cues with an analysis of the mechanisms of differences in response to those cues and the ecological implications of changes in individual traits. In nature, the integration of all levels of information is further complicated by the fact that the developmental environment is more complex than one single changing cue, the phenotype is more than one particular trait, and the selective environment presents more than one ecological challenge.

## Material and methods

### Experimental animals

We used a large outbred laboratory colony of *Bicyclus anynana* butterflies [20]. Hundreds of eggs collected from this stock were distributed over three climate-controlled rooms (70% relative humidity, 12:12 hour light/dark cycle) differing in ambient temperature (±0.5°C). We chose temperatures that simulate the conditions of the natural dry (19°C) and wet (27°C) seasons, and an intermediate temperature (23°C). Larvae were fed *ad libitum* with young maize plants. Pre-pupae were collected daily and pupation times determined (± 15 minutes) by time-lapse digital photography (Canon EOD 100 camera, GT time-lapse remote control). Female pupae from each temperature were split into three experimental groups: non-injected, injected with control solution, and injected with hormone solution (see below). We started with 28 to 70 per temperature per treatment but final sample sizes were smaller for some groups (for example, due to mortality associated with early hormone injections; see below). For non-injected butterflies, we obtained 33 females reared at 19°C, 31 at 23°C, and 38 at 27°C.

### Image analysis of target traits

The ventral surface of the right forewing and hindwing, and the dorsal surface of the forewing of the eclosed females with undamaged wings were photographed (Leica DC200 digital camera) under a binocular microscope (Leica MZ12) with controlled light and 10× magnification. We included a ruler for conversion from pixels to millimeters and a color reference card (QPcard 201) for background correction. The resulting images were analyzed with a custom macro image processing system using an ImageJ-based open-source Fiji software package [48]. For each trait, areas were calculated by a threshold method in which the image was first converted to black and white and values of intensity under or above user-established threshold values were chosen. The measurements of the white central areas of the smaller more anterior eyespots on the forewing (dorsal and ventral, traits 1 and 3, respectively) and hindwing (trait 5) were excluded because of high measurement error. In total, we measured 19 traits characterizing the area of wings and of various color pattern components (Figure 2). We also counted the number of white eyespot centers on the dorsal surface of the hindwing of the non-injected butterflies [34]. Note that the number of females obtained for each treatment is not necessarily equal to the number of measurements available for the 19 traits. This is because not all traits could be measured in all females (for example, in cases of some damaged wings). Final sample sizes for all traits in all experimental groups are given in Additional file 1 for the non-injected individuals and Additional file 2 for early and late injections, respectively.

### Hormone injections

For each temperature, we had two injection treatments: ‘hormone’ for injection of a solution of 20E, the biologically-active form of ecdysone [49] and ‘control’ for injection of the same volume of solvent only. Because the duration of pupal stage varies with temperature, as does the dynamics of ecdysone titers [21], we used % of the duration of the pupal stage when choosing the injection time points. Injections were done on pupae at two stages corresponding to different phases of the natural ecdysone dynamics [21]: ‘early’ (at 3% of the total pupal development time) before ecdysone levels start to increase and ‘late’ (at 16% of the total pupal development time) corresponding to the ascending phase of the ecdysone levels (Figure 1A). Pupae were injected (10 μL Hamilton syringe with a 0.3 mm gauge needle) on the left side in the region of the fifth abdominal segment with 3 μL of 0.25 μg 20E (Sigma-Aldrich: St Louis, MO, USA hormone stock solution 1 mg/ml in 100% ethanol) in insect Ringer’s buffer (Merck: Darmstadt, Germany) with vital red artificial coloring (Fluka (Sigma–Aldrich group): Buchs, Switzerland). This hormone concentration was chosen to obtain an optimal balance between hormonal effects and pupal survival (compare with [23]). After injection, pupae were placed back at their respective rearing temperature until emergence, and adults were frozen (−20°C) until wing analysis. The number of females phenotyped for early injections of control:hormone were 32:19 for 19°C, 29:8 for 23°C and 35:7 for 27°C. For late injections, these numbers were 32:32 for 19°C, 23:30 for 23°C and 34:32 for 27°C. Because not all traits could be measured for each female, the final number of measurements for each trait can be different and are shown in Additional file 2 for early and late injections. Smaller sample sizes for early hormone injections are due to higher mortality associated with that treatment.

### Hormone titers

We injected female pupae reared at 19°C and 27°C with hormone and control solutions at 3% of the duration of pupal stage, and measured internal 20E at 3.5% or at 8.5% of total pupal development time. For that, we extracted 50 μl of hemolymph from each of four pupae per treatment and time point, and measured 20E levels using the ACE enzyme immunoassay (Cayman Chemical Co., Ann Arbor, MI, USA) following the manufacturer's instructions. Briefly, samples were extracted from individual pupae by homogenization followed by addition of 200 μl of 70% methanol. The homogenates were dried using a rotary evaporator at room temperature and dissolved in assay buffer. Calibration curves were generated using commercially available 20E (Sigma; 0.5 μg/μl in 100% ethanol). Absorbance for controls, standards, and hemolymph samples was measured by spectrophotometry at a wavelength of 405 nm (VICTOR Multilabel Plate Reader). Note that this hormone quantification method can detect concentrations down to a minimum concentration of 7.8 pg/μl, which is below the detection level of the method used previously to characterize the titer dynamics displayed in Figure 1A [21].

### Immunohistochemistry

Antibody staining of pupal wings was performed as described in [20] using a custom antibody against *B. anynana* EcR [43] obtained from ProteinTech Group, Chicago, IL, USA (peptide within region common to all isoforms: CWDVADVNSAQPPPVFDHASDL) at a final dilution of 1:50 (after testing a range of concentrations). The antibody was tested together with other antibodies to assess: 1) specificity by comparing its localization with the *Manduca* anti-EcR (we observed similar patterns but with less background for the *B. anynana*-specific antibody); 2) detection of the active form of EcR by comparing its localization with that of the known downstream EcR target Broad; 3) association with the eyespot field and intra-cellular localization by comparing with the localization of 4′,6-diamidino-2-phenylindole (DAPI). We also detected EcR in younger ‘clearer’ tissues (larval wings) in order to confirm the intra-cellular localization of this antibody. We performed stainings of wings dissected from multiple pupae and covering 6% to 30% of pupal duration for each of the two extreme rearing temperatures 27°C and 19°C. The primary anti-EcR antibody was detected with Alexa Fluor 594 anti-rabbit (Molecular Probes, Invitrogen AG, Basel, Switzerland) and images were collected on a Leica DMIRE2, Leica SP5 confocal laser scanning and Nikon Eclipse TE2000-S Screening microscopes.

### Statistical analysis of effects of developmental temperature on wing traits

All data analyses were done using the *R* statistics package [50] and *Mathematica* software package [51]. We tested for the effect of temperature on wing traits of non-injected individuals (Figure 3) using ANOVA with *temperature* as a factor (three levels: 19°C, 23°C, 27°C) and for wing pattern traits 1 to 8, using the respective *wing area* as covariate with the model *trait ~ wing area + temperature*. Trait areas were used untransformed or log10 transformed to meet the Shapiro-Wilk normality test (*P* ≥0.05). When *temperature* was found to have a significant effect on trait values (*P* <0.01), we did *post-hoc* comparisons between pairs of temperatures using lsmeans [see Additional file 1]. To test for the effect of temperature on the number of white pupils on the dorsal surface of the hindwing we used an ANOVA with a Chi-square test and a quasi-Poisson distribution. We tested the model *pupil nr ~ temperature*, using *temperature* as a factor (three levels: 19°C, 23°C, 27°C).

### Statistical analysis of differences in hormone titers

We tested for the effect of temperature and injection treatment on the levels of 20E at two developmental time points (Figure 1B) using the model 20E ~ time point + temperature * injection. We first confirmed that the residuals showed no significant departure from normality (Shapiro-Wilk test) or from homogeneity of variances (Fligner-Killeen test). We then used ANOVA to test for the effect on levels of 20E of *time point* (factor with two levels: 3.5% and 8.5%), *temperature* (factor with levels 19°C and 27°C), *injection* (factor with two levels: hormone and control) and the interaction temperature*injection. Because there was no significant effect of *time point*, we did pairwise comparisons between *temperature* and *injection* groups using Tukey’s honest significance tests [see Additional file 4].

### Statistical analyses of the effects of hormone manipulations on wing traits

We tested for the effect of hormone injections, done at different temperatures and at different developmental time points, on wing traits (Figure 4) using core and confirmatory tests in a series of steps. Details of the analyses are shown in Additional file 2. To facilitate between-trait comparisons, we rescaled raw trait measurements to an identical (0 to 1) range. This was done for each of 114 groups (3 temperatures × 2 injection treatments × 2 time points × 19 traits) by setting the minimum trait value to 0 and the maximum value to 1 and rescaling intermediate values proportionally. We then checked the normal distribution of the rescaled trait values in each group (Jarque-Bera test, alpha = 0.01). For normally distributed values, we used a two-tailed T test to compare control and hormone treatment means for each trait, temperature and time point. For the one non-normally distributed group values (hindwing area, trait 10, after early injection at 27°C), we used a two-tailed Mann–Whitney U test to compare control and hormone medians. We used the false discovery rate procedure [52] with alpha = 0.05 to determine the contextual significance of each of the 57 *P*-values obtained per injection time point.

To take into account differences across treatments in sample size and, particularly, the reduced sample sizes in the early hormone injection groups [23], we carried out an extra validation statistical analysis. We combined two types of resampling techniques [53]: (1) bootstrap (a good method to estimate population parameter differences from small samples); and (2) permutation tests to determine the significance (*P*-values) of the parameter differences (or displacements) obtained via the bootstrap distributions. We performed a bootstrap-based estimation of the displacement of mean/median for each group by resampling 1,000 times from the original distributions of trait values (keeping sample size with replacement). Because the bootstrap distributions did not depart significantly from normality (Jarque-Bera test, alpha = 0.01), we used the mean of that distribution as the estimator of mean displacement (difference) between control and hormone-injected groups. We then used permutation tests to compare differences between control and hormone injections (for each trait, temperature and time point) assessed from the original dataset with those from the resampled dataset. For each of the 57 pairs (19 traits × 3 temperatures × 2 time points) of control and hormone groups, we computed the difference between their original means, and then estimated mean difference 1,000 times from resampled data as follows (note that only means were used on the basis that no bootstrap distribution for the previous goal departs significantly from normality): 1) we merged the two distributions (control with hormone values) into a single distribution; 2) 1,000 times, we divided the values in this distribution into two groups of the same sizes as the original control and hormone groups; 3) we calculated the mean difference between these groups; 4) we thus produced a list of 1,000 mean differences (in absolute value); and 5) we calculated a *P*-value for our original comparison of control versus hormone means as the proportion of those 1,000 values that is different from the original mean difference divided by 1,000 (two-tailed test). The *P*-values obtained were also subjected to the false discovery rate procedure [52] with alpha = 0.05 to determine the contextual significance of each of the 57 *P*-values obtained per injection time point. We compared both sets of results obtained from the core test (k-sample t-test or Mann–Whitney as appropriate) and from permutation tests and found them to be not in conflict [see Additional file 2].

## Additional files

Additional file 1:
**Results S1.** Summary of ANOVA results to test the effect of temperature on wing traits of non-injected individuals (compare with Figure 3). Additional file 2:
**Results S2.** Summary of statistical analyses for wing trait values upon early and late control and hormone injections. This file supports results in Figure 4 and contains Tables S1 (for early injections) and S2 (for late injections) displaying sample sizes, mean and standard error of the re-scaled trait values, difference between hormone and control values (before and after bootstrap), as well as the *p*-values for the statistical significance of those differences. Additional file 3: Figure S1. Hormone injection phenocopies effects of higher developmental temperature. This figure shows the extent to which hormone manipulations at lower temperatures increase trait areas to levels characteristic of higher temperatures. Additional file 4:
**Results S3.** Summary of ANOVA results to test the effect of temperature and injection treatment on the levels of 20E at two developmental time points (compare with Figure 1B). Additional file 5: Figure S2. Patterns of coordinated response to external and internal signals. This figure illustrates which traits responded in concert and in contrast to either the temperature treatment (compare with Figure 3) or the hormone manipulations (compare with Figure 4) and shows in detail the findings summarized in Figure 5. Additional file 6:
**Results S4.** Principal components analysis (PCA) for variation in eyespot traits, separately for non-injected individuals (compare with Figure 3) and for hormone manipulations (compare with Figure 4). This file contains the material and methods, figures S3 and S4, as well as results and discussion of the PCA.
