# Supplementary material for: Adaptive developmental plasticity: Compartmentalized responses to environmental cues and to corresponding internal signals provide phenotypic flexibility
Source: BMC Biol. 2014 Nov 21;12:97. doi: 10.1186/s12915-014-0097-x (PMC4275937; doi:10.1186/s12915-014-0097-x)
Supplement: Additional file 1: — Results S1. Summary of ANOVA results to test the effect of temperature on wing traits of non-injected individuals (compare with Figure 3). [file 12915_2014_97_MOESM1_ESM.pdf]

Additional file 1: Summary of ANOVA results to test the effect of temperature on wing traits of un-injected individuals (cf. Figure 3).

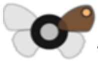

TRAIT 1b:
aov(log10(AreaBlackRingAnteriorEyespotFWD)~WingAreaFW+as.factor(Temperature))
(19C N=30, 23C N=29, 27C N=29)

ANOVA (Type II tests)

	Sum Sq	Df	F value	Pr(>F)
WingAreaFW	0.0011	1	0.051	0.822
Temperature	0.8778	2	19.734	9.43e-08 ***
Residuals	1.8681	84		

lsmeans (alpha = 0.01)

Trt	lsmean	M
27	0.09922766	a
23	0.03682455	a
19	-0.13633344	b

	estimate	SE	df	t.ratio	p.value
19 - 23	-0.1731580	0.03884212	84	-4.45800	0.00007***
19 - 27	-0.2355611	0.03896278	84	-6.04580	0.00000***
23 - 27	-0.0624031	0.03923870	84	-1.59035	0.25542

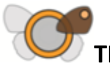

TRAIT 1g:
aov(AreaGoldenRingAnteriorEyespotFWD~WingAreaFW+as.factor(Temperature))
(19C N=30, 23C N=29, 27C N=30)

ANOVA (Type II tests)

	Sum Sq	Df	F value	Pr(>F)
WingAreaFW	0.000	1	0.005	0.671
Temperature	3.072	2	16.350	9.82e-07 ***
Residuals	7.984	85		

lsmeans (alpha = 0.01)

Trt	lsmean	M
27	1.438314	a
23	1.235659	a
19	0.985723	b

	estimate	SE	df	t.ratio	p.value
19 - 23	-0.2499356	0.07982724	85	-3.13096	0.00669***
19 - 27	-0.4525907	0.07929234	85	-5.70787	0.00000***
23 - 27	-0.2026551	0.07989256	85	-2.53660	0.03445

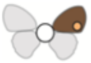

TRAIT 2w:
aov(AreaWhiteRingPosteriorEyespotFWD~WingAreaFW+as.factor(Temperature))
(19C N=30, 23C N=25, 27C N=30)

ANOVA (Type II tests)

	Sum Sq	Df	F value	Pr(>F)
WingAreaFW	0.0193	1	3.384	0.0695
Temperature	0.0134	2	1.174	0.3142
Residuals	0.4622	81		

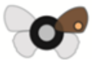

TRAIT 2b:
aov(log10(AreaBlackRingPosteriorEyespotFWD)~WingAreaFW+as.factor(Temperature))
(19C N=30, 23C N=24, 27C N=28)

ANOVA (Type II tests)

	Sum Sq	Df	F value	Pr(>F)
WingAreaFW	0.0222	1	2.678	0.1057
Temperature	0.0742	2	4.478	0.0144
Residuals	0.6465	78		

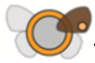

TRAIT 2g:
aov(log10(AreaGoldenRingPosteriorEyespotFWD)~WingAreaFW+as.factor(Temperature))
(19C N=30, 23C N=29, 27C N=30)

ANOVA (Type II tests)

	Sum Sq	Df	F value	Pr(>F)
WingAreaFW	0.0760	1	7.231	0.00862 **
Temperature	0.0388	1	1.846	0.062973
Residuals	0.8930	85		

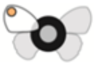

TRAIT 3b:
aov(AreaBlackRingAnteriorEyespotFWV~WingAreaFW+as.factor(Temperature))
(19C N=30, 23C N=30, 27C N=30)

ANOVA (Type II tests)

	Sum Sq	Df	F value	Pr(>F)
WingAreaFW	0.282	1	4.029	0.0479
Temperature	4.170	2	29.841	1.44e-10 ***
Residuals	6.009	86		

lsmeans (alpha = 0.01)

Trt	lsmean	M
27	1.0628883	a
23	1.0362378	a
19	0.5916139	b

	estimate	SE	df	t.ratio	p.value
19 - 23	-0.44462397	0.06839210	86	-6.50110	0.00000***
19 - 27	-0.47127440	0.06853885	86	-6.87602	0.00000***
23 - 27	-0.02665044	0.06827739	86	-0.39033	0.91953

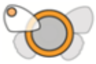

TRAIT 3g:
aov(log10(AreaGoldenRingAnteriorEyespotFWV)~WingAreaFW+as.factor(Temperature))
(19C N=30, 23C N=30, 27C N=30)

ANOVA (Type II tests)

	Sum Sq	Df	F value	Pr(>F)
WingAreaFW	0.0009	1	0.045	0.833
Temperature	1.6427	2	3.626	6.36e-13 ***
Residuals	1.7826	86		

lsmeans (alpha = 0.01)

Trt	lsmean	M
27	0.1845944	a
23	0.1313089	a
19	-0.1261672	b

	estimate	SE	df	t.ratio	p.value
19 - 23	-0.25747612	0.03725006	86	-6.91210	0.00000***
19 - 27	-0.31076160	0.03732999	86	-8.32472	0.00000***
23 - 27	-0.05328548	0.03718758	86	-1.43288	0.32868

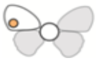

TRAIT 4w:

aov(AreaWhiteRingPosteriorEyespotFWV~WingAreaFW+as.factor(Temperature))

(19C N=30, 23C N=30, 27C N=30)

ANOVA (Type II tests)

	Sum Sq	Df	F value	Pr(>F)
WingAreaFW	0.1164	1	13.936	0.000339 ***
Temperature	0.1399	2	8.372	0.000476 ***
Residuals	0.7185	86		

lsmeans (alpha = 0.01)

Trt	lsmean	M
27	0.5466376	a
23	0.5331569	a
19	0.4567188	b

	estimate	SE	df	t.ratio	p.value
19 - 23	-0.07643811	0.02364909	86	-3.23218	0.00491**
19 - 27	-0.08991874	0.02369983	86	-3.79407	0.00080***
23 - 27	-0.01348062	0.02360942	86	-0.57098	0.83590

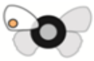

TRAIT 4b:

aov(log10(AreaBlackRingPostFWV)~WingAreaFW+as.factor(Temperature))

(19C N=30, 23C N=25, 27C N=25)

ANOVA (Type II tests)

	Sum Sq	Df	F value	Pr(>F)
WingAreaFW	0.01337	1	3.987	0.04944
Temperature	0.03396	2	5.064	0.00862 **
Residuals	0.25482	76		

lsmeans (alpha = 0.01)

Trt	lsmean	M
27	0.5466376	a
23	0.5331569	a,b
19	0.4567188	b

	estimate	SE	df	t.ratio	p.value
19 - 23	-0.037039513	0.01583647	76	-2.33887	0.05650
19 - 27	-0.046868386	0.01569311	76	-2.98656	0.01000**
23 - 27	-0.009828872	0.01645487	76	-0.59732	0.82197

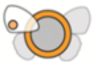

TRAIT 4g:

aov(AreaGoldenRingPostFWV~WingAreaFW+as.factor(Temperature))

(19C N=30, 23C N=30, 27C N=30)

ANOVA (Type II tests)

	Sum Sq	Df	F value	Pr(>F)
WingAreaFW	13.48	1	8.523	0.004476 **
Temperature	28.82	2	9.109	0.000258 ***
Residuals	136.03	86		

lsmeans (alpha = 0.01)

Trt	lsmean	M
27	6.971511	a
23	6.970646	a
19	5.765887	b

	estimate	SE	df	t.ratio	p.value
19 - 23	-1.2047594104	0.3254036	86	-3.70235	0.00109**
19 - 27	-1.2056244015	0.3261019	86	-3.69708	0.00111**
23 - 27	-0.0008649912	0.3248578	86	-0.00266	1.00000

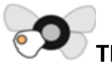

TRAIT 5b:

aov(AreaBlackRingSecondEyespotHW~WingAreaHW+as.factor(Temperature))

(19C N=27, 23C N=30, 27C N=30)

ANOVA (Type II tests)

	Sum Sq	Df	F value	Pr(>F)
WingAreaHW	0.1635	1	9.579	0.00268**
Temperature	1.3050	2	38.225	1.71e-12 ***
Residuals	1.4168	83		

lsmeans (alpha = 0.01)

Trt	lsmean	M
27	0.5074119	a
23	0.4002296	b
19	0.1882168	c

	estimate	SE	df	t.ratio	p.value
19 - 23	-0.2120128	0.03547793	83	-5.97591	0.00000***
19 - 27	-0.3191951	0.03699319	83	-8.62848	0.00000***
23 - 27	-0.1071823	0.03415631	83	-3.13800	0.00659**

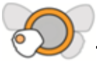

TRAIT 5g:

aov(AreaGoldenRingSecondEyespotHW~WingAreaHW+as.factor(Temperature))

(19C N=27, 23C N=30, 27C N=30)

ANOVA (Type II tests)

	Sum Sq	Df	F value	Pr(>F)
WingAreaHW	0.0125	1	0.96	0.33
Temperature	0.7239	2	27.79	5.77e-10 ***
Residuals	1.0810	83		

lsmeans (alpha = 0.01)

Trt	lsmean	M
27	0.5688483	a
23	0.5551609	a
19	0.3538453	b

	estimate	SE	df	t.ratio	p.value
19 - 23	-0.20131557	0.03098912	83	-6.49633	0.00000***
19 - 27	-0.21500299	0.03231266	83	-6.65383	0.00000***
23 - 27	-0.01368742	0.02983472	83	-0.45877	0.89063

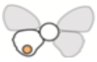

TRAIT 6w:

aov(log10(AreaWhiteRingFifthEyespotHW)~WingAreaHW+as.factor(Temperature))

(19C N=27, 23C N=30, 27C N=30)

ANOVA (Type II tests)

	Sum Sq	Df	F value	Pr(>F)
WingAreaHW	0.323	1	13.80	0.000368***
Temperature	3.736	2	79.71	<2e-16 ***
Residuals	1.945	83		

lsmeans (alpha = 0.01)

Trt	lsmean	M
27	-0.9347217	a
23	-1.1240441	b
19	-1.4762416	c

	estimate	SE	df	t.ratio	p.value
19 - 23	-0.3521975	0.04157154	83	-8.47208	0e+00***
19 - 27	-0.5415199	0.04334706	83	-12.49266	0e+00***
23 - 27	-0.1893224	0.04002292	83	-4.73035	3e-05***

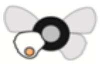

TRAIT 6b:

aov(log10(AreaBlackRingFifthEyespotHW)~WingAreaHW+as.factor(Temperature))

(19C N=27, 23C N=30, 27C N=30)

ANOVA (Type II tests)

	Sum Sq	Df	F value	Pr(>F)
WingAreaHW	0.0005	1	0.062	0.804
Temperature	1.1680	2	67.657	< 2e-16 ***
Residuals	0.7165	83		

lsmeans (alpha = 0.01)

Trt	lsmean	M
27	0.4940979	a
23	0.4073894	b
19	0.1953067	c

	estimate	SE	df	t.ratio	p.value
19 - 23	-0.2120827	0.02522881	83	-8.40637	0.00000***
19 - 27	-0.2987912	0.02630633	83	-11.35815	0.00000***
23 - 27	-0.0867085	0.02428899	83	-3.56987	0.00171**

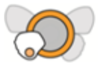

TRAIT 6g:

aov(AreaGoldenRingFifthEyespotHW~WingAreaHW+as.factor(Temperature))

(19C N=27, 23C N=30, 27C N=30)

ANOVA (Type II tests)

	Sum Sq	Df	F value	Pr(>F)
WingAreaHW	0.734	1	5.285	0.024
Temperature	14.037	2	50.569	4.35e-15 ***
Residuals	11.519	83		

lsmeans (alpha = 0.01)

Trt	lsmean	M
27	2.235777	a
23	1.781466	b
19	1.176153	c

	estimate	SE	df	t.ratio	p.value
19 - 23	-0.6053131	0.10116118	83	-5.98365	0e+00***
19 - 27	-1.0596246	0.10548178	83	-10.04557	0e+00***
23 - 27	-0.4543116	0.09739275	83	-4.66474	3e-05***

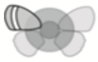

TRAIT 7:

aov(BandAreaFWV~WingAreaFW+as.factor(Temperature))

(19C N=30, 23C N=30, 27C N=30)

ANOVA (Type II tests)

	Sum Sq	Df	F value	Pr(>F)
WingAreaFW	0.424	1	3.593	0.0614
Temperature	7.223	2	30.602	9.18e-11 ***
Residuals	10.149	86		

lsmeans (alpha = 0.01)

Trt	lsmean	M
27	2.078562	a
23	1.691148	b
19	1.383329	c

	estimate	SE	df	t.ratio	p.value
19 - 23	-0.3078194	0.08888264	86	-3.46321	0.00238**
19 - 27	-0.6952333	0.08907336	86	-7.80518	0.00000***
23 - 27	-0.3874139	0.08873356	86	-4.36604	0.00010***

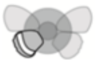

TRAIT 8:

aov(log10(BandAreaHW)~WingAreaHW+as.factor(Temperature))

(19C N=27, 23C N=30, 27C N=30)

ANOVA (Type II tests)

	Sum Sq	Df	F value	Pr(>F)
WingAreaHW	0.0052	1	0.51	0.477
Temperature	0.2415	2	11.78	3.13e-05 ***
Residuals	0.8507	83		

lsmeans (alpha = 0.01)

Trt	lsmean	M
27	0.18183176	a
23	0.09264033	b
19	0.04660616	b

	estimate	SE	df	t.ratio	p.value
19 - 23	-0.04603417	0.02749073	83	-1.67453	0.22103
19 - 27	-0.13522561	0.02866485	83	-4.71747	0.00003***
23 - 27	-0.08919143	0.02646665	83	-3.36996	0.00325**

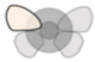

TRAIT 9:

aov(WingAreaFW~as.factor(Temperature))

(19C N=31, 23C N=31, 27C N=31)

ANOVA (Type II tests)

	Sum Sq	Df	F value	Pr(>F)
Temperature	973	2	0.631	0.535
Residuals	69435	90		

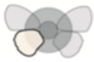

TRAIT 10:

aov(WingAreaHW~as.factor(Temperature))

(19C N=27, 23C N=30, 27C N=30)

ANOVA (Type II tests)

	Sum Sq	Df	F value	Pr(>F)
Temperature	11630	2	5.878	0.00408**
Residuals	83105	84		

lsmeans (alpha = 0.01)

trt	lsmean	M
19	384.8245	a
23	368.0986	ab
27	356.2872	b

	estimate	SE	df	t.ratio	p.value
19 - 23	16.72595	8.343891	84	2.00457	0.11737
19 - 27	28.53732	8.343891	84	3.42015	0.00276**
23 - 27	11.81137	8.121347	84	1.45436	0.31812

Nr White Pupils on dorsal surface of hindwing

aov(log10(NFociHWD)~as.factor(Temperature))

(19C N=33, 23C N=30, 27C N=38)

ANOVA (Type II tests)

	Df	Deviance	Resid. Df	Resid. Dev	Pr(>Chi)
NULL			99	42.937	
Temperature	2	1.8949	97	41.042	0.09819
