# Supplementary material for: Adaptive developmental plasticity: Compartmentalized responses to environmental cues and to corresponding internal signals provide phenotypic flexibility
Source: BMC Biol. 2014 Nov 21;12:97. doi: 10.1186/s12915-014-0097-x (PMC4275937; doi:10.1186/s12915-014-0097-x)
Supplement: Additional file 2: — Results S2. Summary of statistical analyses for wing trait values upon early and late control and hormone injections. This file supports results in Figure 4 and contains Tables S1 (for early injections) and S2 (for late injections) displaying sample sizes, mean and standard error of the re-scaled trait values, difference between hormone and control values (before and after bootstrap), as well as the p-values for the statistical significance of those differences. [file 12915_2014_97_MOESM2_ESM.pdf]

Table S1. Summary of statistical analyses for trait values upon early control and hormone injections

Trait	Temp	Control injection			Hormone injection			H-C	p-value	H-C	StError	p-value
		N	Mean	StError	N	Mean	StError			bootstrap	bootstrap	permut.
1b	19C	30	25.352	3.134	9	21.468	5.101	-3.884	0.609	-3.475	0.184	0.658
	23C	29	41.137	2.910	6	28.413	7.628	-12.724	0.045	-14.965	0.248	0.980
	27C	29	56.520	3.198	7	61.170	6.184	4.649	0.965	3.673	0.245	0.508
1g	19C	30	37.547	2.913	9	35.236	4.379	-2.311	0.913	0.615	0.152	0.473
	23C	29	45.675	3.095	6	51.704	5.287	6.028	0.635	5.433	0.222	0.331
	27C	29	52.791	2.916	7	43.414	7.200	-9.377	0.589	-9.377	0.342	0.674
2w	19C	30	23.757	1.914	9	22.775	4.998	-0.982	0.509	-3.211	0.160	0.768
	23C	29	42.049	3.188	6	54.258	10.278	12.209	0.496	4.896	0.318	0.262
	27C	29	36.798	2.434	7	39.808	2.751	3.010	0.975	-0.233	0.109	0.523
2b	19C	30	44.869	2.307	9	32.555	5.955	-12.315	0.154	-7.519	0.191	0.910
	23C	29	51.034	2.782	6	44.858	7.041	-6.176	0.656	-6.095	0.337	0.677
	27C	29	53.983	3.057	7	55.121	5.411	1.139	0.426	0.931	0.259	0.789
2g	19C	30	46.180	2.372	9	37.326	4.768	-8.855	0.236	-5.897	0.157	0.881
	23C	29	49.496	2.869	6	64.665	7.251	15.169	0.082	12.749	0.214	0.036
	27C	29	48.342	2.573	7	57.473	5.755	9.132	0.150	8.554	0.187	0.081
3b	**19C**	**30**	**21.517**	**2.843**	**11**	**39.572**	**2.967**	**18.055**	**0.003**	**15.875**	**0.133**	**0.002**
	23C	29	42.086	2.706	8	47.337	5.182	5.251	0.855	4.642	0.236	0.535
	27C	28	49.281	3.386	7	49.235	5.858	-0.046	0.880	-0.804	0.198	0.576
3g	**19C**	**30**	**15.792**	**1.327**	**11**	**36.251**	**3.563**	**20.459**	**0.000**	**19.567**	**0.113**	**0**
	**23C**	**29**	**28.118**	**2.251**	**8**	**55.474**	**7.704**	**27.357**	**0.007**	**27.638**	**0.237**	**0**
	27C	28	37.949	3.885	7	42.764	6.680	4.815	0.620	5.512	0.261	0.310
4w	19C	29	40.356	1.697	11	39.611	2.332	-0.745	0.559	-1.831	0.091	0.676
	23C	29	59.004	2.177	8	57.652	6.089	-1.353	0.859	-1.353	0.230	0.562
	27C	28	60.586	3.404	7	60.442	3.790	-0.144	0.765	-2.301	0.151	0.657
4b	19C	30	35.424	2.749	11	33.779	3.753	-1.646	0.567	-3.051	0.143	0.723
	23C	29	50.630	2.709	8	40.679	4.881	-9.951	0.207	-7.561	0.159	0.900
	27C	28	50.283	3.409	7	39.327	3.597	-10.957	0.427	-9.960	0.195	0.802
4g	19C	30	27.501	1.960	11	35.776	4.397	8.274	0.031	8.388	0.174	0.019
	23C	29	48.099	2.771	8	53.379	7.085	5.281	0.237	5.316	0.239	0.121
	27C	28	48.251	3.497	7	50.515	7.215	2.264	0.296	8.691	0.240	0.154
5b	**19C[b]**	**30**	**12.875**	**3.072**	**7**	**22.738**	**6.062**	**9.863**	**0.014**	**9.831**	**0.120**	**0.074**
	23C	20	32.406	3.483	6	36.168	5.555	3.762	0.227	4.952	0.273	0.094
	27C	26	42.618	2.516	5	61.387	6.345	18.769	0.488	18.757	0.608	0.243
5g	**19C**	**30**	**29.057**	**3.311**	**7**	**42.009**	**5.321**	**12.952**	**0.000**	**15.056**	**0.114**	**0**
	**23C**	**20**	**44.308**	**3.914**	**6**	**69.234**	**5.282**	**24.926**	**0.000**	**22.446**	**0.179**	**0.001**
	27C	26	53.983	3.349	5	44.246	2.665	-9.737	0.746	-9.737	0.536	0.349
6w	19C	30	9.981	1.384	7	14.258	2.504	4.277	0.113	3.577	0.138	0.009
	23C	20	35.283	2.920	6	33.688	7.952	-1.595	0.454	5.094	0.202	0.212
	27C	26	41.083	3.451	5	46.158	11.995	5.075	0.584	4.688	0.336	0.703
6b	**19C**	**30**	**24.796**	**1.678**	**7**	**35.283**	**3.460**	**10.486**	**0.007**	**10.042**	**0.129**	**0.007**
	23C	20	46.579	2.449	6	50.740	5.885	4.161	0.257	7.367	0.161	0.138
	27C	26	55.761	3.098	5	67.882	11.135	12.121	0.405	6.136	0.158	0.219
6g	**19C**	**30**	**16.257**	**0.674**	**7**	**37.342**	**2.851**	**21.086**	**0.005**	**21.086**	**0.241**	**0**
	**23C**	**20**	**31.997**	**3.202**	**6**	**46.766**	**5.788**	**14.770**	**0.002**	**15.461**	**0.114**	**0.001**
	27C	26	40.029	2.891	5	44.278	7.037	4.248	0.709	3.403	0.171	0.324
7	19C	30	35.351	1.811	11	36.586	3.923	1.236	0.288	6.683	0.205	0.138
	23C	29	62.842	3.135	8	71.665	4.499	8.823	0.616	3.565	0.196	0.318
	27C	27	67.300	3.066	7	68.434	4.529	1.134	0.856	1.182	0.206	0.422
8	**19C**	**30**	**24.640**	**1.276**	**7**	**43.137**	**4.679**	**18.497**	**0.006**	**21.037**	**0.172**	**0**
	23C	20	49.418	2.199	6	53.574	3.119	4.156	0.390	6.832	0.177	0.213
	27C	26	48.662	3.866	5	55.053	4.384	6.392	0.936	0.691	0.212	0.430
9	19C	31	62.597	1.696	11	61.389	5.818	-1.208	0.520	-4.325	0.195	0.729
	23C	29	51.266	3.618	8	53.429	4.767	2.163	0.767	2.382	0.189	0.383
	27C	33	51.175	2.979	7	56.729	6.598	5.554	0.093	7.498	0.130	0.141
10	19C	30	69.516	2.334	7	70.402	2.421	0.886	0.910	-0.679	0.101	0.555
	23C	20	56.737	4.409	6	61.411	7.183	4.674	0.553	5.226	0.253	0.294
	27C[a]	26	56.442	3.390	5	51.984	2.668	-4.458	0.271	-5.413	0.127	0.735

For each trait, we display sample size, mean and standard error of the values re-scaled (cf. Material and Methods, shown here as %) for the control and hormone injections at each temperature. "H-C" is the difference between mean value for hormone and mean value for control. We show difference before and after bootstrap analysis and corresponding p-values (two-tailed t-test; alpha=0.01). *For the only group with non-normally distributed values, we show median and used Mann-Whitney U test. Bold grey cells correspond to statistically significant differences at 5% false discovery rate (same as red symbols in Fig 4).

Table S2. Summary of statistical analyses for trait values upon late control and hormone injections

Trait	Temp	Control injection			Hormone injection			H-C	p-value	H-C	StError	p-value
		N	Mean	StError	N	Mean	StError			bootstrap	bootstrap	permut.
1b	19C	30	34.493	2.981	30	37.604	3.082	3.111	0.443	3.325	0.135	0.237
	23C	20	52.678	4.969	28	57.977	3.486	5.299	0.588	3.552	0.182	0.389
	27C	30	57.789	3.160	30	61.220	3.298	3.430	0.818	1.117	0.141	0.432
1g	19C	30	27.094	2.868	30	40.011	3.056	12.917	0.028	8.477	0.126	0.024
	23C	20	38.098	5.255	28	48.557	3.272	10.459	0.601	8.541	0.200	0.686
	27C	30	54.032	3.342	30	55.303	3.118	1.271	0.891	0.551	0.144	0.413
2w	19C	30	34.739	2.208	30	28.591	2.744	-6.147	0.918	-4.941	0.168	0.479
	23C	20	40.790	3.703	28	42.593	3.154	1.804	0.805	-1.402	0.155	0.682
	27C	30	41.740	3.278	30	44.461	3.008	2.721	0.911	-0.368	0.138	0.590
2b	19C	30	45.034	2.437	30	48.144	2.602	3.109	0.389	3.188	0.117	0.205
	23C	20	56.957	4.841	28	57.895	2.717	0.938	0.814	1.329	0.171	0.526
	27C	30	56.700	2.762	30	56.065	3.454	-0.635	0.504	2.987	0.139	0.260
2g	19C	30	43.469	2.415	30	49.362	2.877	5.893	0.164	5.299	0.118	0.079
	23C	20	48.058	4.442	28	57.538	2.653	9.480	0.056	9.496	0.156	0.047
	27C	30	54.745	3.024	30	67.310	3.596	12.565	0.044	9.758	0.145	0.023
3b	19C	30	37.004	3.499	30	41.991	2.986	4.987	0.476	3.327	0.139	0.156
	23C	20	50.733	3.376	27	57.374	2.527	6.640	0.287	6.327	0.115	0.295
	27C	30	57.253	2.661	30	60.793	2.486	3.540	0.056	7.035	0.116	0.026
3g	19C	30	23.416	2.406	30	27.495	2.287	4.079	0.191	4.339	0.101	0.091
	23C	20	40.921	4.262	27	42.191	3.241	1.269	0.805	1.481	0.164	0.401
	27C	30	49.272	3.702	30	54.726	3.749	5.454	0.947	2.250	0.167	0.314
4w	19C	30	47.138	1.816	30	56.776	2.261	9.638	0.223	3.431	0.091	0.129
	23C	20	59.716	3.799	27	61.566	2.268	1.849	0.836	0.829	0.141	0.300
	27C	30	60.657	2.600	30	63.578	2.783	2.921	0.194	5.069	0.121	0.085
4b	19C	30	42.637	2.572	30	45.243	2.554	2.606	0.404	3.068	0.111	0.185
	23C	20	50.458	3.856	27	51.996	2.487	1.538	0.624	2.139	0.145	0.298
	27C	30	47.845	1.971	30	49.713	2.550	1.868	0.297	4.010	0.103	0.096
4g	19C	30	30.232	2.761	30	36.533	2.530	6.300	0.098	6.135	0.116	0.064
	23C	20	49.448	4.166	27	48.702	2.765	-0.746	0.940	-0.592	0.156	0.547
	27C	30	52.338	2.776	30	53.467	3.562	1.128	0.469	3.354	0.141	0.226
5b	19C	29	20.198	2.242	30	21.421	1.712	1.223	0.758	0.709	0.115	0.616
	23C	18	40.409	3.210	22	47.378	3.731	6.969	0.096	6.485	0.144	0.052
	27C	30	54.057	3.639	30	55.191	3.886	1.134	0.732	-2.080	0.171	0.638
5g	19C	29	33.674	2.376	30	37.638	2.325	3.965	0.174	4.644	0.108	0.082
	**23C**	**18**	**31.338**	**3.341**	**22**	**51.285**	**3.702**	**19.947**	**0.001**	**17.875**	**0.152**	**1.20E-05**
	27C	30	61.065	3.469	30	62.331	2.083	1.266	0.853	0.656	0.124	0.386
6w	19C	29	12.518	1.356	29	12.422	1.166	-0.097	0.414	-1.447	0.057	0.709
	23C	18	28.420	2.390	22	28.130	1.957	-0.290	0.689	-1.255	0.095	0.642
	27C	30	53.359	3.144	30	43.668	2.314	-9.691	0.052	-7.749	0.124	0.984
6b	19C	29	33.857	2.509	29	31.528	1.865	-2.329	0.323	3.003	0.095	0.156
	23C	18	57.756	3.621	22	57.792	2.653	0.036	0.582	2.486	0.137	0.309
	27C	30	63.704	3.116	30	65.001	2.276	1.297	0.528	2.523	0.121	0.263
6g	19C	29	22.487	1.767	30	22.361	1.398	-0.126	0.622	1.249	0.070	0.299
	23C	18	33.985	2.033	22	39.795	2.557	5.811	0.173	4.517	0.102	0.078
	27C	30	50.387	2.964	30	50.803	2.116	0.417	0.313	-3.711	0.113	0.856
7	19C	30	37.480	3.472	30	31.256	2.212	-6.224	0.305	-4.374	0.128	0.848
	23C	20	42.907	3.108	27	63.656	3.671	20.750	0.020	12.147	0.155	0.007
	27C	30	69.332	3.652	30	69.914	2.515	0.582	0.235	5.526	0.141	0.112
8	19C	29	33.681	2.543	30	20.554	2.107	-13.128	0.032	-12.448	0.122	0.985
	23C	18	40.956	5.596	22	44.164	3.973	3.208	0.871	1.143	0.209	0.542
	27C	30	53.131	3.593	30	53.505	2.711	0.374	0.762	-1.317	0.145	0.619
9	19C	30	66.695	3.227	30	76.021	2.442	9.326	0.080	7.107	0.128	0.039
	23C	20	52.332	3.402	28	57.404	2.364	5.073	0.042	8.392	0.132	0.044
	27C	31	56.104	2.621	30	57.500	2.977	1.396	0.355	3.735	0.120	0.191
10	19C	29	74.437	2.463	30	78.303	2.337	3.866	0.280	3.027	0.161	0.128
	23C	18	66.735	3.897	22	63.286	2.788	-3.449	0.844	1.000	0.145	0.444
	27C	30	61.877	2.358	30	60.305	2.338	-1.572	0.724	1.126	0.100	0.365

For each trait, we display sample size, mean and standard error of the values re-scaled (cf. Material and Methods, shown here as %) for the control and hormone injections at each temperature. “H-C” is the difference between mean value for hormone and mean value for control. We show difference before and after bootstrap analysis and corresponding p-values (two-tailed t-test; alpha=0.01). Bold grey cells correspond to statistically significant differences at 5% false discovery rate (same as red symbols in Fig 4).
