# Supplementary material for: Adaptive developmental plasticity: Compartmentalized responses to environmental cues and to corresponding internal signals provide phenotypic flexibility
Source: BMC Biol. 2014 Nov 21;12:97. doi: 10.1186/s12915-014-0097-x (PMC4275937; doi:10.1186/s12915-014-0097-x)
Supplement: Additional file 3: Figure S1. — Hormone injection phenocopies effects of higher developmental temperature. This figure shows the extent to which hormone manipulations at lower temperatures increase trait areas to levels characteristic of higher temperatures. [file 12915_2014_97_MOESM3_ESM.pdf]

Additional file 3: Hormone injection phenocopies effects of higher developmental temperature.

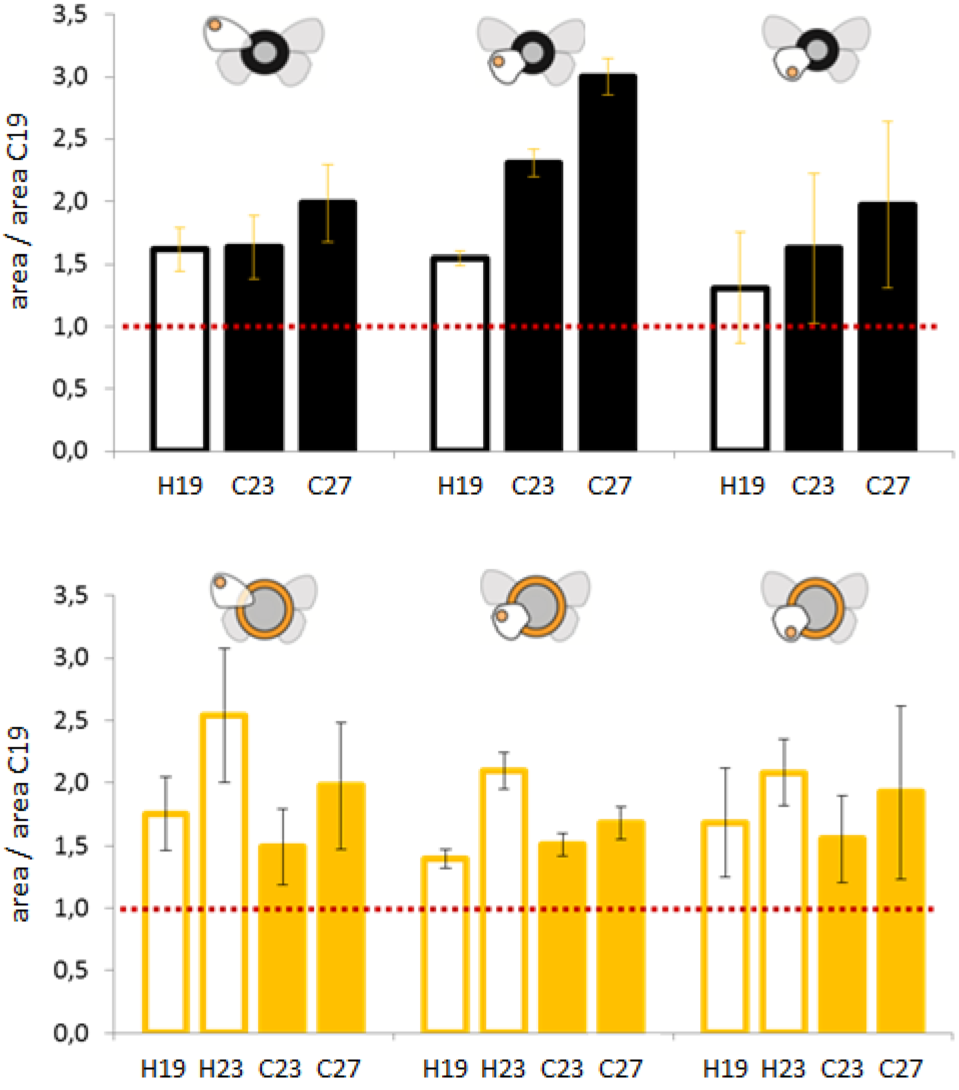

Figure S1: Hormone injection phenocopies the effect of higher developmental temperature. For each of the traits (icon cf. Figure 2) that changed significantly with early hormone injection (cf. Figure 4), bar height represents the ratio between the trait average for each treatment (C or H for control or hormone, respectively, and 19, 23, or 27 for the three rearing temperatures; labels in X axis) and that of control-injected individuals reared at 19°C (C19; cf. Y-axis). Error bars correspond to standard deviations.
