# Supplementary material for: Adaptive developmental plasticity: Compartmentalized responses to environmental cues and to corresponding internal signals provide phenotypic flexibility
Source: BMC Biol. 2014 Nov 21;12:97. doi: 10.1186/s12915-014-0097-x (PMC4275937; doi:10.1186/s12915-014-0097-x)
Supplement: Additional file 4: — Results S3. Summary of ANOVA results to test the effect of temperature and injection treatment on the levels of 20E at two developmental time points (compare with Figure 1B). [file 12915_2014_97_MOESM4_ESM.pdf]

Additional file 4: Summary of ANOVA results to test the effect of temperature and injection treatment on the levels of 20E at two developmental time points (cf. Figure 1B).

model<- lm(20E ~ TP + Temperature * Injection)

Shapiro-Wilk normality test
W = 0.9502, p-value = 0.1464

Fligner-Killeen test of homogeneity of variances
Fligner-Killeen:med chi-squared = 1.7552, df = 1, p-value = 0.1852

Anova (Response: 20E)

	Sum Sq	Df	F value	Pr(>F)
TP	0.8	1	0.0258	0.8735524
Temperature	416.2	1	13.8484	0.0009206 ***
Injection	3441.0	1	114.5008	3.258e-11 ***
Temperature:Injection	110.3	1	3.6697	0.0660575 .
Residuals	811.4	27		

HSD.test (model,c("Temperature","Injection")

Treatment	Means	Comparison
alpha=0.001		
27:Hormone	47.20847	a
19:Hormone	43.70869	a
27:Control	30.18182	b
19:Control	19.25634	b
alpha=0.005		
27:Hormone	47.20847	a
19:Hormone	43.70869	a
27:Control	30.18182	b
19:Control	19.25634	c
alpha=0.01		
27:Hormone	47.20847	a
19:Hormone	43.70869	a
27:Control	30.18182	b
19:Control	19.25634	c
alpha=0.05		
27:Hormone	47.20847	a
19:Hormone	43.70869	a
27:Control	30.18182	b
19:Control	19.25634	c
