# Supplementary material for: Adaptive developmental plasticity: Compartmentalized responses to environmental cues and to corresponding internal signals provide phenotypic flexibility
Source: BMC Biol. 2014 Nov 21;12:97. doi: 10.1186/s12915-014-0097-x (PMC4275937; doi:10.1186/s12915-014-0097-x)
Supplement: Additional file 5: Figure S2. — Patterns of coordinated response to external and internal signals. This figure illustrates which traits responded in concert and in contrast to either the temperature treatment (compare with Figure 3) or the hormone manipulations (compare with Figure 4) and shows in detail the findings summarized in Figure 5. [file 12915_2014_97_MOESM5_ESM.pdf]

Additional file 5: Patterns of coordinated response to external and internal signals.

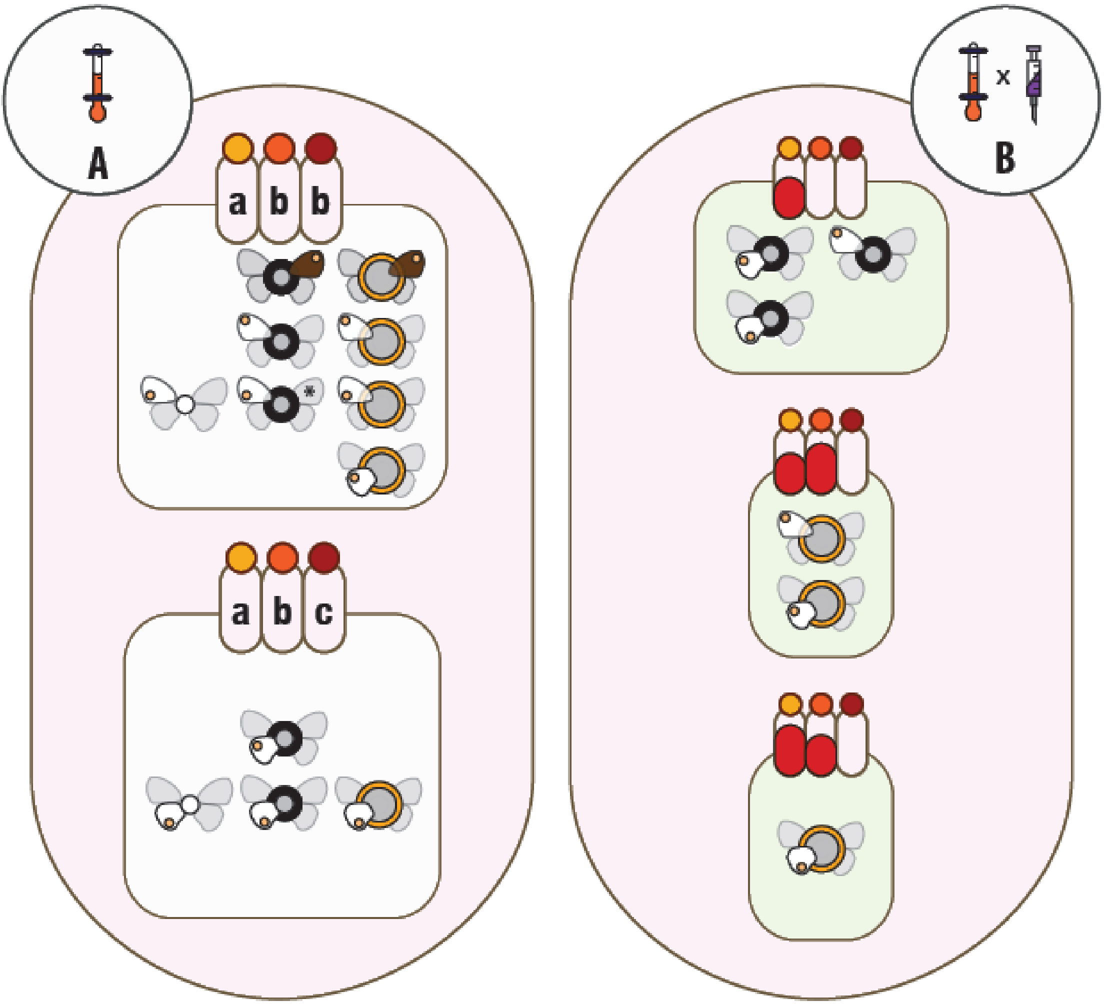

Figure S2: Patterns of coordinated response to external and internal signals. Each box includes all eyespot traits that responded in a similar manner to differences in developmental temperature **(A)** and to hormone injections **(B)**. The three insets at the top of each box represent each of the three experimental temperatures. In panel **(A)**, the letters inside the insets are the same as those in Figure 3 relative to the pairwise comparisons on the reaction norms. They illustrate whether the trait areas are (different letters) or not (same letter) significantly different across temperatures. We see two types of responses: traits sizes at 19˚C < 23˚C ~ 27˚C (inset a-b-b), and trait sizes at 19˚C < 23˚C < 27˚C (inset a-b-c). The * on trait 2b (Figure 2) corresponds to one exception where values are statistically different between 19˚C and 27˚C and do not differ between either of these extreme temperatures and 23˚C (inset a-ab-b in Figure 3). In panel **(B)**, the red areas inside the insets illustrate the magnitude of statistically significant differences between control and hormone treatments at each of the temperatures (cf. Figure 4). We find three types of response to hormone injections: effect only for 19˚C, effect at 19˚C > 23˚C, and effect at 19°C < 23°C. The patterns of response to temperature contrast fore- and hindwing (with one exception) while those for hormone manipulations contrast black and golden color rings. The light green background corresponds to that used in Figure 4 for early hormone manipulations.
