# Supplementary material for: Adaptive developmental plasticity: Compartmentalized responses to environmental cues and to corresponding internal signals provide phenotypic flexibility
Source: BMC Biol. 2014 Nov 21;12:97. doi: 10.1186/s12915-014-0097-x (PMC4275937; doi:10.1186/s12915-014-0097-x)
Supplement: Additional file 6: — Results S4. Principal components analysis (PCA) for variation in eyespot traits, separately for non-injected individuals (compare with Figure 3) and for hormone manipulations (compare with Figure 4). This file contains the material and methods, figures S3 and S4, as well as results and discussion of the PCA. [file 12915_2014_97_MOESM6_ESM.pdf]

Additional file 6: PCA for variation in eyespot traits, separately for non-injected inividuals (cf. Figure 3) and for hormone manipulations (cf. Figure 4).

MATERIAL AND METHODS

We used Principal Component Analysis (PCA) technique to explore and describe the patterns of variation for the 15 eyespot rings [1]. In order to handle missing values, we used the FactoMineR [2] and missMDA [3] packages for R. We analyzed separately the eyespot trait values for non-injected individuals from the three rearing temperatures (Figure S3), and the early and late, control- and hormone-injected individuals at the temperature (Figure S4). For each of the analysis, we present the tables with the loadings for the first three principal components (Dims) and the plots of the data distribution along those Dims. The PCA analysis was run on trait area/wing area, to account for differences in wing size.

1. Jolliffe IT: Principal Component Analysis. Springer, New York: Springer; 1986.
2. Lê S, Josse J, Husson F: FactorMineR: An R package for multivariate analysis. J Stat Softw 2008, 25:1–18.
3. Husson F, Josse J: missMDA - Handling missing values with/in multivariate data analysis (principal component methods). R package version 1.2, 2010.

TRAIT	Dim 1	Dim 2	Dim 3
1b	0.734	0.339	-0.261
1g	0.777	0.276	-0.323
2w	0.526	0.631	0.111
2b	0.621	0.655	-0.175
2g	0.560	0.611	-0.274
3b	0.829	-0.271	0.061
3g	0.842	-0.234	0.114
4w	0.668	0.209	0.421
4b	0.659	0.173	0.455
4g	0.731	0.132	0.494
5b	0.838	-0.327	-0.134
5g	0.769	-0.378	-0.117
6w	0.759	-0.358	-0.078
6b	0.838	-0.418	-0.127
6g	0.824	-0.346	-0.092
**Eigenvalue**	8.1799	2.2835	0.9989
**% variation**	54.5	15.2	6.7

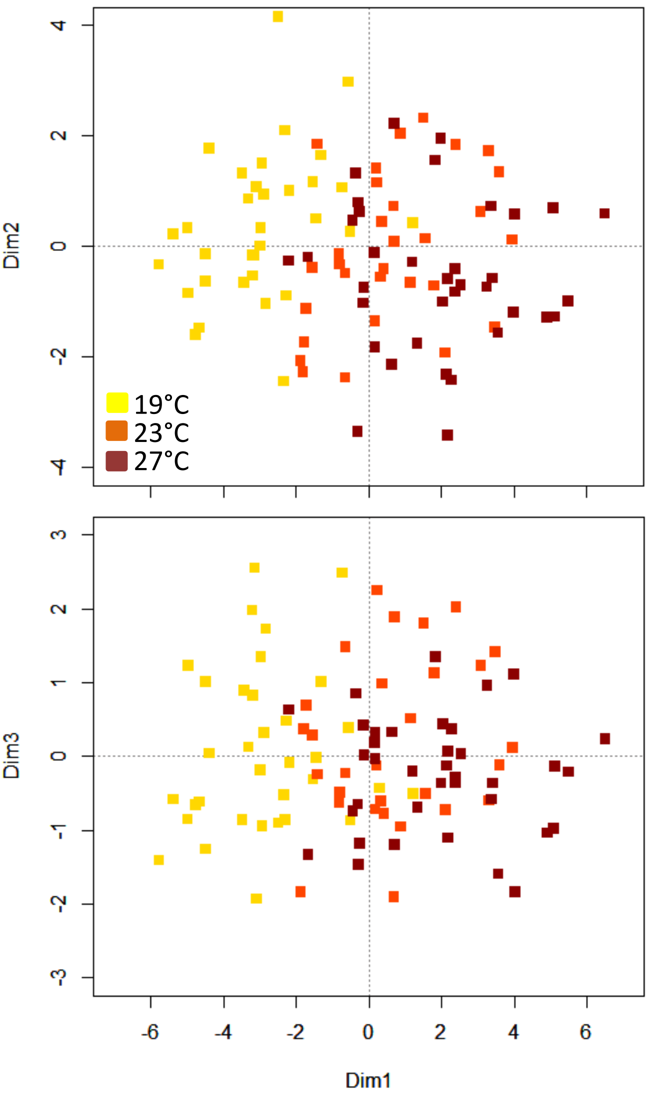

Figure S3: PCA for variation in eyespot traits with developmental temperature (cf. data represented in Figure 3). The table represents the loadings for the first three principal components (called Dims). The plots represent the scores for all measured individuals along Dims 1-3, separated by rearing temperature .

TRAIT	Dim 1	Dim 2	Dim 3
1b	0.768	0.274	0.309
1g	0.698	0.314	0.429
2w	0.734	0.479	-0.162
2b	0.712	0.519	0.117
2g	0.594	0.557	0.244
3b	0.808	-0.295	-0.003
3g	0.816	-0.341	0.093
4w	0.778	0.286	-0.401
4b	0.771	0.266	-0.317
4g	0.781	0.191	-0.289
5b	0.782	-0.435	0.074
5g	0.725	-0.474	0.116
6w	0.775	-0.340	-0.064
6b	0.863	-0.349	-0.044
6g	0.804	-0.364	0.011
**Eigenvalue**	8.7334	2.1602	0.7591
**% variation**	58.2	14.4	5.1

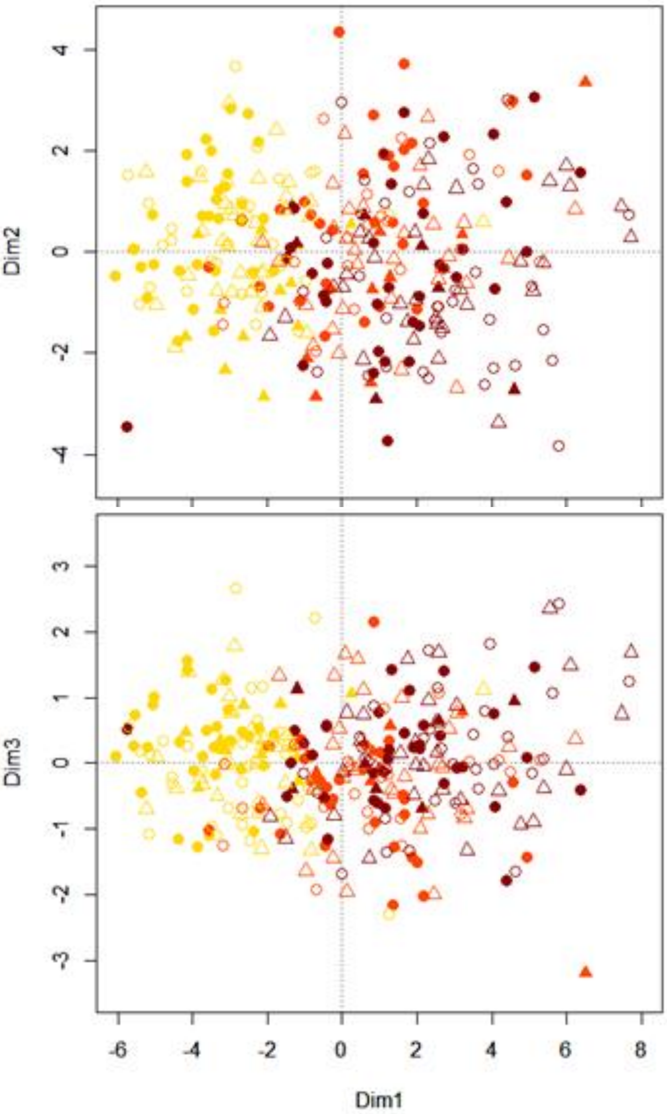

Figure S4: PCA for variation in eyespot traits with temperature and hormone manipulations (cf. data represented in Figure 4). The table represents the loadings for the first three principal components (called Dims). The plots represent the scores for all measured individuals along Dims 1-3, separated by rearing temperature (symbol color cf. Fig. S3), injection treatment (Δ hormone, ○ control) and time point (filled or empty symbol for early and late injections, respectively).

The PCA describing the patterns of variation for eyespot ring colors (trait area/wing area) from different temperatures enabled us to reduce the variation to three main dimensions (Dims), together accounting for 76.4% of the variation in our data (Figure S3). Dim1 appears to describe overall trait size, with all traits contributing equally (all coefficients have the same sign and approximate value; Table) and separating temperatures (Plots). Dim2 appears to reflect the extent of temperature-responsiveness (cf. Figure 3): negative loadings for the most responsive traits (eyespots 3, 5 and 6), intermediate positive values for less responsive traits (eyespots 1 and 4), and highest positive values for non-responsive traits (eyespot 2). Dim3 is harder to interpret. It contrasts forewing (positive loadings) and hindwing (negative loadings) for traits on the ventral surface (as in our Figure 5), but not for the dorsal surface.

The PCA describing the patterns of variation for the 15 traits (trait area/wing area) from different temperatures, treatments and time points has enabled us to reduce the variation to three Dims together accounting for 77.7% of the variation in our data (Figure S4). Dim1 has similar loadings for all traits and clearly separates temperatures. Dim2 appears to reflect the extent of temperature-responsiveness as described above. Dim3 is, again, harder to interpret. For ventral patterns, it largely contrasts black and white eyespot rings (with the most extreme negative loadings for non-hormone responsive traits of eyespot 4, including golden ring) versus golden areas with positive loadings.
